# Rice phytochelatin synthases OsPCS1 and OsPCS2 make different contributions to cadmium and arsenic tolerance

**DOI:** 10.1002/pld3.34

**Published:** 2018-01-09

**Authors:** Shinichi Yamazaki, Yosuke Ueda, Aya Mukai, Kumiko Ochiai, Toru Matoh

**Affiliations:** ^1^ Graduate School of Agriculture Kyoto University Kyoto Japan

**Keywords:** arsenic, cadmium, glutathione, OsPCS, phytochelatin, rice

## Abstract

Cadmium (Cd) and arsenic (As) pollution in paddy soil and their accumulation in rice (*Oryza sativa*) pose serious threats to human health. Rice internally detoxifies these toxic metal and metalloid to some extent, resulting in their accumulation within the edible parts. However, the mechanisms of Cd and As detoxification in rice have been poorly elucidated. Plants synthesize thiol‐rich metal‐chelating peptides, termed phytochelatins (PCs). We characterized rice PC synthase (PCS) and investigated its contribution to Cd and As tolerance in rice. We identified two *PCS* homolog genes, *OsPCS1* and *OsPCS2*, in the rice genome. The expression of *OsPCS1* was upregulated by As(III) stress in the roots but that of *OsPCS2* was not significantly affected. The expression level of *OsPCS2* was higher than that of *OsPCS1* in the shoots and roots. Recombinant OsPCS1 and OsPCS2 proteins differed in their metal activation. OsPCS1 was more strongly activated by As(III) than by Cd; however, OsPCS2 was more strongly activated by Cd than by As(III). Genetically engineered plants having their *OsPCS2* expression silenced via RNA interference (*OsPCS2* RNAi) contained less PCs and more glutathione (GSH), a substrate of PC synthesis, than wild‐type plants, although there was no significant difference in *OsPCS1* RNAi plants. *OsPCS2* RNAi plants were sensitive to As(III) stress, but Cd tolerance was little affected. On the other hand, treatment with buthionine sulfoximine, an inhibitor of GSH biosynthesis, significantly decreased Cd and As tolerance of rice seedlings. These findings indicate that OsPCS2 is a major isozyme controlling PC synthesis, and that PCs are important for As tolerance in rice. However, PC synthesis may make a smaller contribution to Cd tolerance in rice, and GSH plays crucial roles, not only as a substrate of PC synthesis.

## 1 INTRODUCTION

Cadmium (Cd) and arsenic (As) are a heavy metal and metalloid that are toxic to living organisms including humans and plants. The contamination of paddy fields by Cd and As is an agricultural problem in some areas of South‐East and South Asia (Brammer & Ravenscroft, 2009; Sriprachote, Kanyawongha, Ochiai, & Matoh, 2012; Williams et al., 2009), and exposure to Cd and As through the consumption of contaminated rice (*Oryza sativa*), a staple food for Asian populations, could pose a serious health hazard (Clemens, Aarts, Thomine, & Verbruggen, 2013; Meharg et al., 2009). In soils with a low level of pollution, rice plants do not show obvious growth inhibition caused by Cd or As (Abedin, Cotter‐Howells, & Meharg, 2002; Ito & Iimura, 1976), although these toxic metal/metalloid accumulate in the shoot and grain. Therefore, it is important to understand the mechanisms of Cd and As detoxification in rice.

When exposed to Cd and As, plants and some microorganisms produce a class of thiol‐rich peptides with the general structure of (γGlu‐Cys)_n_‐Gly, termed phytochelatins (PCs) (Schmöger, Oven, & Grill, 2000; Toppi & Gabbrielli, 1999; Verbruggen, Hermans, & Schat, 2009). Peptides are enzymatically synthesized from glutathione (GSH; γGlu‐Cys‐Gly) in a reaction catalyzed by PC synthase (PCS). *PCS* genes are constitutively expressed in plants, and the PCS enzyme is post‐translationally activated by the presence of heavy metals/metalloids such as Cd and As (Cobbett, 2000; Vatamaniuk, Mari, Lu, & Rea, 2000). PCs bind Cd and As(III) in the cytosol, and PC‐Cd and PC‐As complexes are then sequestered into vacuoles. Complexation and sequestration are crucial for Cd and As tolerance in thale cress (*Arabidopsis thaliana*). *PCS* genes were first isolated from *Arabidopsis*, wheat (*Triticum aestivum*), and fission yeast (*Schizosaccharomyces pombe*) (Clemens, Kim, Neumann, & Schroeder, 1999; Ha et al., 1999; Vatamaniuk, Mari, Lu, & Rea, 1999), and the *Arabidopsis pcs1* mutant that lacked the ability to synthesize PC was shown to be hypersensitive to Cd and As (Ha et al., 1999; Howden, Goldsbrough, Andersen, & Cobbett, 1995). Two ATP‐binding cassettes (ABC) transporters, AtABCC1 and AtABCC2, were identified to mediate the vacuolar transport of PC‐As and PC‐Cd complexes, and the *abcc1 abcc2* double‐knockout *Arabidopsis* mutant was also highly sensitive to Cd and As (Park et al., 2012; Song et al., 2010). The formation of PCs in response to Cd stress is a ubiquitous feature in the plant kingdom as revealed by a survey of a large number of plant species belonging to Bryophyta, Pteridophyta, and Spermatophyta (Gekeler, Grill, Winnacker, & Zenk, 1989). It is now clear that *PCS* genes are present in all higher plants. In several plant species, there are at least two *PCS* genes in their genome (Clemens, 2006). Rice also has two *PCS* genes on chromosomes 5 and 6, termed *OsPCS1* and *OsPCS2*. Concerning *OsPCS*, it was reported that silencing *OsPCS1* by RNA interference (RNAi) using a seed‐specific promoter reduced Cd accumulation in rice seeds (Li, Guo, Xu, & Ma, 2007). Recently, Das, Bhattacharya, Bhattacharyya, and Maiti (2017) also reported that seed‐specific downregulation of *OsPCS1* and *OsPCS2* decreased Cd and As contents in the grain. These reports indicate that PC synthesis contributes to Cd and As accumulation in rice grain. However, the physiological roles in and contributions of the two OsPCS isozymes to Cd and As tolerance of rice plants have not yet been investigated.

The bioavailability of Cd and As varies between upland crops and paddy rice because the metal and metalloid solubility is dependent on the redox status of the rhizosphere. Under aerobic soil conditions, the solubility of Cd is high in aerobic soil, whereas the majority of As is present in its oxidized, less mobile arsenate As(V) form, so that plants easily take up Cd; however, As uptake is suppressed (Arao, Kawasaki, Baba, Mori, & Matsumoto, 2009). In contrast, under anaerobic conditions such as flooded paddy soil, Cd forms an insoluble sulfide, whereas As(V) is reduced to its highly soluble arsenite As(III) form (Ito & Iimura, 1976; Masscheleyn, Delaune, & PatrickJr, 1991; Yamane, 1989). Thus, paddy rice tends to take up more As than upland plants owing to its soil environment. Furthermore, rice is more efficient than other cereal crops in As(III) uptake and translocation, probably because As(III) is taken up by the rice root through its silicon transport pathway (Ma et al., 2008; Su, McGrath, & Zhao, 2010). Such redox states with opposing effects in the soil and different uptake capacities might affect subcellular responses to Cd and As among plant species. However, Cd and As detoxification mechanisms in rice have been poorly elucidated.

In this study, we characterized the enzymatic and physiological properties of OsPCS and investigated the contribution of PC synthesis to Cd and As tolerance in rice. We found that silencing the *OsPCS* gene increased the As sensitivity of rice seedlings, whereas it had a small effect on Cd tolerance. Our findings suggest that PC synthesis is important for As tolerance in rice, whereas it seems to make a little contribution to Cd tolerance, which contrasts with the case in upland plants such as *Arabidopsis*.

## 2 MATERIALS AND METHODS

### 2.1 Plant material and growth conditions

The *japonica* rice cultivar Nipponbare and transgenic plants of RNAi generated from this cultivar were used in this study. Seeds were soaked for 3 days at 30°C in distilled water supplied with 0.3% (w/v) fungicide (Trifmine; Nippon Soda Co., Ltd.). Seeds were then sown on plastic meshes floating on the culture solution. Seedlings were hydroponically grown in an artificial climate incubator at 30°C and 80% relative humidity with a 12‐hr light period. The control culture solution contained 1 mmol/L (NH_4_)_2_SO_4_, 0.5 mmol/L KCl, 0.25 mmol/L KH_2_PO_4_, 0.5 mmol/L CaCl_2_, and 0.5 mmol/L MgCl_2_ for macronutrients and 37.7 mg/L EDTA‐FeNa, 28.6 mg/L H_3_BO_3_, 1.81 mg/L MnCl_2_・4H_2_O, 0.08 mg/L CuSO_4_・5H_2_O, 0.22 mg/L ZnSO_4_・7H_2_O, and 0.1 mg/L (NH_4_)_6_Mo_7_O_24_・4H_2_O for micronutrients. At harvest, roots and basal stems were washed with distilled water for 30 s and gently blotted on paper towels.

### 2.2 Growth experiment under Cd or As toxicity

Wild‐type (WT) and RNAi plants were hydroponically grown in a culture solution containing 10 μmol/L CdSO_4_ or 20 μmol/L NaAsO_2_ and the control culture solution. Seedlings were harvested, and the shoot and root lengths were measured 10 days after sowing.

For the growth experiment of *OsPCS2* RNAi plants under Cd toxicity, the WT and RNAi plants were hydroponically grown for 7 days in the control culture solution. Seven‐day‐old seedlings of similar sizes were selected and transferred to culture solutions containing 0, 3, 10, or 30 μmol/L CdSO_4_. A plate with 20 holes was set on a 1‐L plastic container, and one plant was put into each hole supported by a piece of polyurethane sponge. Seven days after transfer, plants were harvested and shoot and root lengths were measured.

### 2.3 Buthionine sulfoximine treatment

Seven‐day‐old seedlings of Nipponbare grown in the control culture solution were transferred to glass vials, one seedling per vial, containing 50 ml of the culture solution supplemented or not with toxic metals [10 μmol/L Cd or 10 μmol/L As(III)] either alone or in combination with 0.5 mmol/L buthionine sulfoximine (BSO), an inhibitor of GSH synthesis. The treatment solution was renewed 4 days after transfer. At 7 days after transfer, plants were harvested and shoot and root lengths, as well as fresh weights of whole plants, were determined.

### 2.4 Homology search and sequence analysis of PCS genes

Two transcript sequences (*OsPCS1*#4 and *OsPCS2*#2) were obtained from a database, and five transcript sequences (*OsPCS1*#1, #2, and #3 and *OsPCS2*#1 and #3) were newly identified in this study. The transcript variants were numbered in the order of the open reading frame (ORF) length from the longest to shortest. First, rice *PCS* gene candidates *OsPCS1* [AF439787 (*OsPCS1*#4)] and *OsPCS2* [AK071754 (*OsPCS2*#2), and AK071958] were obtained by a Basic Local Alignment Search Tool (BLAST) search of the Rice Annotation Project database (RAP‐DB) using an amino acid sequence of *Arabidopsis* PCS (AtPCS1) as a query. Subsequently, a BLAST search of the expressed sequence tag (EST) database of the National Center for Biotechnology Information was conducted using the *OsPCS1*#4 transcript sequence as a query, and eight cDNA clones (CB646798, CK083842, CB646799, CI275800, CI126777, C96924, CI067086, and CI226673) were obtained. We estimated a full‐length transcript sequence [LC192427 (*OsPCS1*#1)] from a comparison between the identified cDNA fragments and a genomic sequence (Figure S1a). In *OsPCS2*, AK071754 (*OsPCS2*#2) corresponds to the N‐terminal half of AtPCS1, and AK071958, which does not contain an initiation codon, corresponds to the C‐terminal half. A full‐length transcript sequence [LC192430 (*OsPCS2*#1)] was estimated from a comparison between the identified transcript sequences and the genomic sequence (Figure S1b). Then, the estimated sequences of *OsPCS1*#1 and *OsPCS2*#1 were confirmed by sequencing PCR amplified fragments. Primer sets for the amplification were PCS1.seq2F and PCS1.seqR for the *OsPCS1*#1 fragment and PCS2.seqF and PCS2.seq1R for the *OsPCS2*#1 fragment (Table S3). Template cDNA was synthesized as described below from the root total RNA of Nipponbare grown in the control culture solution. PCR amplification was carried out using Blend Taq DNA polymerase (TOYOBO). The amplified products were purified using a Mag Extractor (TOYOBO) and then sequenced (FASMAC).

Other shorter transcripts [LC192428 (*OsPCS1*#2), LC192429 (*OsPCS1*#3), and LC192431 (*OsPCS2*#3)] were found during the steps in cloning *OsPCS* gene fragments into an expression vector for the recombinant protein analysis. Sequence analyses of inserted DNA fragments generated using a primer set PCS1.proF and PCS1‐2.3.R revealed the presence of LC192428 (*OsPCS1*#2) and LC192429 (*OsPCS1*#3) besides *OsPCS1*#1. Similarly, sequencing inserted fragments generated using a primer set PCS2.proF and PCS2‐1.proR revealed the presence of LC192431 (*OsPCS2*#3), which had a premature stop codon, besides *OsPCS2*#1. The GT‐AG rule applies in all splice sites of these transcripts (Figure S1).

### 2.5 Gene expression analysis

Expression levels of the multiple transcripts were examined in 10‐day‐old rice seedlings hydroponically grown under 10 μmol/L CdSO_4_ or 10 μmol/L NaAsO_2_ stress as well as in control plants grown without toxic metals. Three plants were bulked, and total RNA was extracted from shoots and roots using the RNeasy Plant Mini Kit (QIAGEN). Genomic DNA in the extracted sample was digested with recombinant DNase I (TaKaRa Bio). Single‐stranded cDNA was synthesized using an oligo (dT)_20_ primer and ReverTra Ace reverse transcriptase (TOYOBO). Gene expression levels were analyzed by quantitative RT‐PCR performed with a THUNDERBIRD SYBR qPCR Mix (TOYOBO). The geometric mean of the expression levels of *ubiquitin* and *actin* was used as the internal standard. For the analysis of multiple transcript expression, expression levels were calibrated by the amplification efficiency of each primer pair.

For the analysis of *OsPCS1/2* expression in RNAi plants, plants were hydroponically grown for four weeks in the control culture solution. The culture solution was renewed once a week. Total RNA was extracted from the shoots and roots of individual plants. The sequences of primers used in the experiment are shown in Table S3.

### 2.6 Preparation and purification of recombinant proteins

Full‐length ORF sequences of *OsPCS1* and *OsPCS2* were amplified by PCR using Prime Star DNA polymerase (TaKaRa Bio) and the primer set, PCS1.proF and PCS1‐1.proR or PCS2.proF and PCS2‐1.proR (Table S3). The reverse primers contained a recognition sequence for SbfI (GGACGTCC) at the 5′ end. The amplified fragment was subcloned into the pMAL‐c5X vector (New England Biolabs) following the manual's instructions. The recombinant maltose‐binding protein (MBP) fusion protein was expressed in the *Escherichia coli* strain NEB Express (ER2523; New England Biolabs) and purified by amylose resin affinity chromatography. Protein purification was confirmed by SDS‐PAGE.

### 2.7 PCS activity assay

The assay solution contained the final concentrations of 10 mmol/L GSH, 200 mmol/L HEPES‐KOH buffer pH 8.0, and 50 μmol/L metal salt (MnCl_2_, NiSO_4_, CoCl_2_, ZnSO_4_, Na_2_MoO_4_, Na_2_HAsO_4_, CuSO_4_, NaAsO_2_, or CdSO_4_). The solution was pre‐incubated at 35°C for 15 min, and the reaction was started by the addition of purified MBP‐OsPCS1 (15 μg/ml) or MBP‐OsPCS2 (5 μg/ml). The reaction was carried out at 35°C for 60 min and was stopped by the addition of trifluoroacetic acid (TFA) at a final concentration of 1% (v/v).

In the assay with different concentrations of Cd or As(III), the assay solution contained 10, 20, 50, 100, or 200 μmol/L NaAsO_2_ or CdSO_4_ and the reaction was carried out for 20 min. Other conditions were the same as mentioned above.

### 2.8 Quantification of thiol peptides

Concentrations of thiol peptides in rice plants were quantified as described by Sneller et al. (2000) with some modification. Shoot and root samples were freeze‐dried and pulverized using a ball mill. Thiol peptides were extracted from 10 mg of the powdered sample by adding 250 μl of 0.1% (v/v) TFA containing 6.3 mmol/L diethylenetriaminepentaacetic acid (DTPA). After centrifugation at 4°C, 125 μl of the supernatant was collected and mixed with 225 μl of 200 mmol/L HEPES buffer pH 8.2 containing 6.3 mmol/L DTPA. To this solution, 10 μl of 50 mmol/L monobromobimane (mBBr) dissolved in dimethyl sulfoxide was added, and a derivatization reaction was performed for 1 hr at room temperature in the dark. The reaction was stopped by the addition of 150 μl of 1 mol/L methanesulfonic acid. The sample solution was kept on ice in the dark until analysis.

For the quantification of PCs synthesized by recombinant proteins, 20 μl of the assay solution after the reaction had been stopped was mixed with 180 μl of 0.1% TFA containing 6.3 mmol/L DTPA. After centrifugation at 4°C, 125 μl of the solution was collected and derivatized, as described above.

A 20‐μl aliquot of the derivatized sample was injected into an HPLC system (LC‐10AS; Shimadzu) equipped with a Hypersil ODS C18 column (5 μm, 12 nm, 4.6 mm × 250 mm; Thermo Fisher Scientific Inc.). The elution was performed at a flow rate of 0.5 ml/min with a linear gradient of methanol and water for 60 min. It was monitored using a fluorescence detector (RF‐10A_XL_; Shimadzu) with the excitation wavelength set at 380 nm and the emission wavelength at 470 nm. Eluent A [10% (v/v) methanol and water containing 0.05% (v/v) TFA] and eluent B (80% methanol and water containing 0.05% TFA) were used in a linear gradient elution with the following time program: At the time of 0 min, eluent B was 28%; 15 min, 30%; 30 min, 40%; 35 min, 45%; 40 min, 58%; 45 min, 75%; 48 min, 90%; 49 min, 100%; 55 min, 28%; and 60 min, 28%.

Artificially synthesized reagents such as GSH (NACALAI TESQUE), PC_2_, PC_4_, hydroxymethyl‐glutathione (hmGSH, γGlu‐Cys‐Ser), γGlu‐Cys (γEC), and γGlu‐Cys‐Glu (γECE) (HiPep Laboratories) were used to identify elution peaks of each thiol peptide. Peaks of PC_3_ and hydroxymethyl‐PC_2_ [hmPC_2_, (γGlu‐Cys)_2_‐Ser] were identified from the chromatogram of an in vitro PCS assay.

### 2.9 Measurement of Cd and As concentrations

Plant materials oven‐dried for 2 days were digested with HNO_3_ and HClO_4_. Digested samples were diluted with 0.1% (v/v) HNO_3_. The Cd concentration was determined using an atomic absorption spectrophotometer (AA‐6300; Shimadzu) with a graphite furnace atomizer (GFA‐EX7i; Shimadzu). The As concentration was determined by inductively coupled plasma atomic emission spectroscopy (SPS1500VR; Seiko Instruments).

### 2.10 Transformation of rice

*OsPCS* RNAi rice plants were produced by *Agrobacterium*‐mediated transformation (Toki et al., 2006). A segment of 317 bp or 327 bp in the 3′ untranslated region specific to *OsPCS1* or *OsPCS2*, respectively, was used for the RNAi trigger. The RNAi trigger sequences have 39.9% identity with each other, as determined by EMBOSS Needle alignment (Figure S3). The trigger sequence was amplified by PCR using Prime Star DNA polymerase (TaKaRa) and the primer set, PCS1.RNAi.F and PCS1.seqR or PCS2.RNAi.F and PCS2.RNAi.R (Table S3). The forward primers contained a CACC sequence at the 5′ end for TOPO cloning. The amplified fragment was cloned into the Gateway pENTR/D‐TOPO cloning vector (Invitrogen). The fragment was subsequently inserted into the pANDA destination vector, which was under the control of a constitutive maize *ubiquitin1* promoter (Miki & Shimamoto, 2004). The RNAi construct was introduced into *Agrobacterium tumefaciens* strain EHA105 (Hood, Gelvin, Melchers, & Hoekema, 1993) and rice (cv. Nipponbare) was transformed, as described by Toki et al. (2006).

## 3 RESULTS

### 3.1 Cloning of rice PCS genes

**Figure 1:**
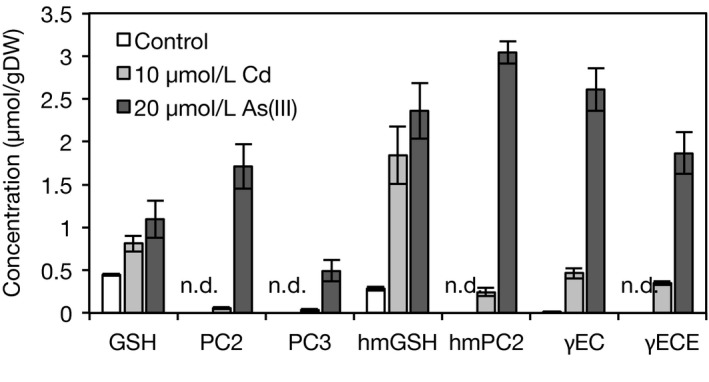
Synthesis of thiol peptides in response to Cd or As(III) stress in rice roots. The 7‐day‐old seedlings of Nipponbare were transferred to a culture solution supplemented with 10 μmol/L Cd or 20 μmol/L As(III) and the control culture solution. Four plants were bulked, and the concentration of thiol peptides in the roots was determined 7 days after transfer. Values are expressed as means ± SE (control: *n* = 3, Cd, As: *n* = 4). GSH, γGlu‐Cys‐Gly; PC_2,3_, (γGlu‐Cys)_2,3_‐Gly; hmGSH, γGlu‐Cys‐Ser; hmPC_2_, (γGlu‐Cys)_2_‐Ser; γEC, γGlu‐Cys; γECE, γGlu‐Cys‐Glu

PCs are known to be important for Cd and As detoxification in higher plants. Here, we first investigated PC synthesis in rice plants. When rice seedlings (cv. Nipponbare) were grown under control conditions, GSH, hmGSH, and less amount of γEC were detected in the shoots and roots, but PC‐like peptides were not detected. In the presence of 10 μmol/L Cd or 20 μmol/L As(III), syntheses of thiol peptides such as GSH, PCs, hmGSH, hydroxymethyl‐PCs (hmPCs, (γGlu‐Cys)_n_‐Ser), γEC, and γECE, were remarkably induced in the roots (Figure 1). The concentration of hmGSH was higher than that of GSH under both Cd and As(III) stresses, although their concentrations were comparable under control conditions. The concentrations of PC‐like peptides, PCs and hmPC_2_, under As(III) stress were much higher than those under Cd stress. In the shoots, Cd and As(III) stresses induced the synthesis of GSH and hmGSH, but PCs and hmPCs were not detected (data not shown). From this result, we hypothesized that PCs were related to Cd and As detoxification in rice, and then we cloned and characterized the rice *PCS* genes.

**Figure 2:**
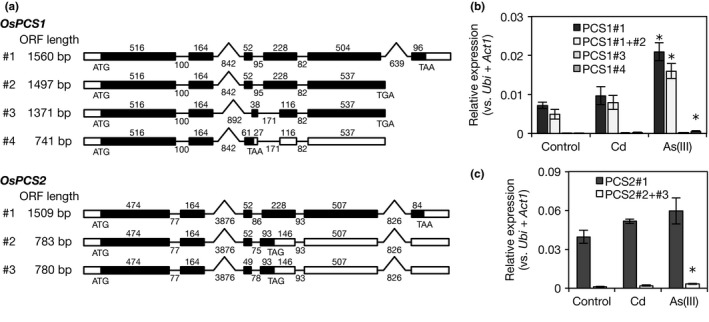
Structure and expression of OsPCS transcripts. (a) Schematic diagrams of the transcriptional variants of *OsPCS1* and *OsPCS2*. Boxes show the exons, and lines show the introns. Open reading frames are indicated as solid boxes and untranslated regions are indicated as open boxes. The numbers above the boxes and below the lines indicate the length of the region. (b, c) Relative expression of *OsPCS1* and *OsPCS2* in roots under control conditions and heavy metal toxicity. The expression level was analyzed by quantitative RT‐PCR in 10‐day‐old roots hydroponically grown under control, Cd stress (10 μmol/L CdSO_4_), or As(III) stress (10 μmol/L NaAsO_2_) condition. *Ubiquitin* and *Actin* were used as internal standards. Differences in PCR amplification efficiency among the primer pairs were corrected using standard curves. Values are expressed as means ± *SE* (*n* = 4). An asterisk (*) shows a significant difference from the control (*p *<* *0.05, Dunnett test)

In a homology search performed using the amino acid sequence of AtPCS1 as a query, the *Os05g0415200* (*OsPCS1*) and *Os06g0102300* (*OsPCS2*) genes were identified in the rice genome. Their ORF sequences in the RAP‐DB (*OsPCS1*: AF439787; *OsPCS2*: AK071754 and AK071958) were too small at only half the length of other plant *PCS* genes. An EST database search and comparison of the hits to a genome sequence of rice suggested probable expression of other transcripts containing longer ORFs (Figure S1). The estimated transcript sequences were verified via sequence analysis using Nipponbare cDNA, and we named them *OsPCS1*#1 (LC192427) and *OsPCS2*#1 (LC192430). Like *PCS* genes in other plant species, the ORF lengths of *OsPCS1*#1 and *OsPCS2*#1 were 1,560 and 1,509 bp, respectively. Their predicted amino acid sequences were 69.6% identical to each other, whereas they were 50% identical to those of the *Arabidopsis* PCS, and 70% identical to those of the wheat PCS (Figure S2 and Table S1). Transcript sequences of half‐length ORF in the RAP‐DB, AF439787 and AK071754, were termed *OsPCS1*#4 and *OsPCS2*#2, respectively. In addition, when we cloned full‐length coding sequences of *OsPCS1*#1 and *OsPCS2*#1, other splice variants containing short‐length ORFs (*OsPCS1*#2 and #3, and *OsPCS2*#3) were isolated (Figure 2a, Figure S1). Predicted amino acid sequences of the N‐terminal half, including the catalytic domain of the PCS enzyme, were identical in OsPCS1#1–4 and OsPCS2#1–3.

### 3.2 Expression of OsPCS transcriptional variants

Alternative splicing in plants has a key regulatory role in modulating gene expression in response to environmental signals, and biotic and abiotic stresses influence alternative splicing (Reddy, 2007; Syed, Kalyna, Marquez, Barta, & Brown, 2012). Therefore, the expression level of each transcriptional variant in rice seedlings was determined via quantitative RT‐PCR using specific primers. Expression levels of the longest ORFs, *OsPCS1*#1 and *OsPCS2*#1, were clearly higher than those of other transcripts (Figure 2b,c). Under As(III) stress, the expression of *OsPCS1* splice variants was upregulated in the roots (Figure 2b), but that in the shoots was not affected (Figure S4). In addition, the expression pattern of *OsPCS1* splice variants was not changed by Cd or As stress in both shoots and roots; the expression level of *OsPCS1*#1 was the highest. The expression of *OsPCS2* remained unchanged under Cd or As(III) stress (Figure 2c; Figure S4). This suggests that *OsPCS1*#1 and *OsPCS2*#1 are expressed as the predominant transcripts.

### 3.3 Enzymatic analysis of recombinant proteins

PCS catalyzes the formation of (γGlu‐Cys)_2_‐Gly (PC_2_) from two GSH molecules, or the formation of PC_n+1_ from GSH and PC_n_. Such catalytic reactions are activated by the presence of various heavy metal/metalloid ions. Cd^2+^, Hg^2+^, and As(III) are the most potent activators of PC synthesis catalyzed by AtPCS1 in vitro (Vatamaniuk et al., 2000).

**Figure 3:**
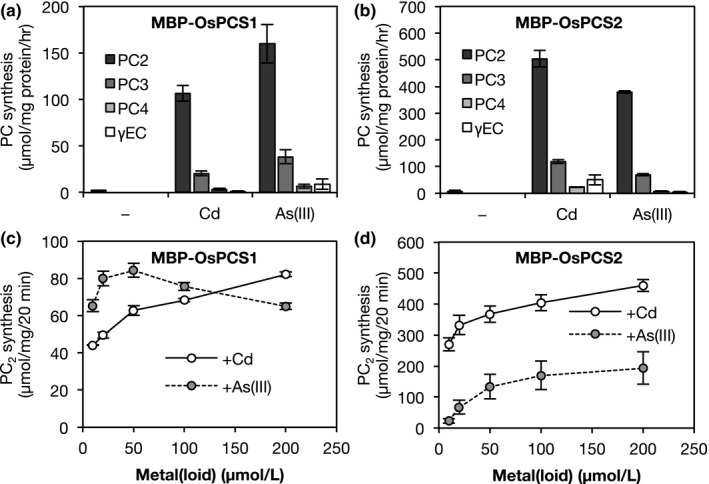
Activation of MBP‐OsPCS by Cd and As(III). (a, b) PC synthesis catalyzed by OsPCS1 (a) and OsPCS2 (b) in the presence of different heavy metals/metalloids. Purified MBP‐OsPCS1 (15 μg/ml) or MBP‐OsPCS2 (5 μg/ml) was incubated for 1 hr in the reaction medium containing 10 mmol/L GSH, 200 mmol/L HEPES‐KOH buffer (pH8.0), and 50 μmol/L Cd^2+^ or As(III) and the nonmetal control. The reaction was stopped by the addition of TFA at a final concentration of 1% (v/v). PC_2–4_ and γEC were derivatized with monobromobimane and quantified by HPLC‐fluorescent detection. (c, d) PC_2_ synthesis catalyzed by OsPCS1 (c) and OsPCS2 (d) at different Cd or As(III) concentrations. The reaction media contained 10, 20, 50, 100, or 200 μmol/L Cd^2+^ or As(III), and the reaction was carried out for 20 min. Values are expressed as means ± SE from three independent experiments

To confirm whether rice PCS homologs could catalyze PC synthesis, we investigated their in vitro enzymatic activity. The PC synthetic activities of MBP‐fused OsPCS1#1 and MBP‐OsPCS2#1 were assayed in the presence of various heavy metal/metalloid ions such as Mn^2+^, Ni^2+^, Co^2+^, Zn^2+^, MoO_4_^2−^, Cu^2+^, As(V), As(III), and Cd^2+^. Among these metals/metalloids, Cd^2+^ and As(III) strongly activated recombinant OsPCS1 and OsPCS2 (Figure S4). Interestingly, Cd^2+^ and As(III) had contrasting activation effects on the two OsPCS isozymes (Figure 3). OsPCS1‐catalyzed PC_2_ synthesis was increased 50‐fold by Cd^2+^ and 70‐fold by As(III) at the concentration of 50 μmol/L compared with the nonmetal control (Figure 3a). In contrast, OsPCS2‐catalyzed PC_2_ synthesis was increased 70‐fold by Cd^2+^ and 50‐fold by As(III) (Figure 3b). In the assay of various concentrations that ranged from 10 to 200 μmol/L, the activation levels of OsPCS2 by Cd^2+^ and As(III) increased in a dose‐dependent manner, and that by Cd^2+^ was always stronger than that by As(III) (Figure 3d). The activation level of OsPCS1 by Cd also increased with increasing Cd concentration; however, that by As(III) reached a peak at 50 μmol/L, above which it gradually decreased (Figure 3c). Therefore, in the low concentration range of 10–50 μmol/L, the activation of OsPCS1 by As(III) was stronger than that by Cd^2+^; however, this was reversed at a high concentration of 200 μmol/L. From this result, we hypothesized that each OsPCS might be related to a specific response to Cd or As in rice plants, and then we investigated the effect of *OsPCS* gene knockdown.

### 3.4 Cd and As sensitivity of OsPCS RNAi plants

**Figure 4:**
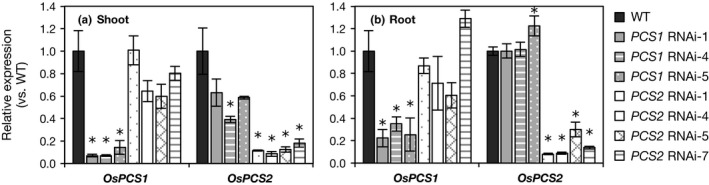
Suppression of OsPCS1 and OsPCS2 expression in RNAi plants. Expression levels in shoots (a) and in roots (b). WT (cv. Nipponbare) and RNAi plants were hydroponically grown for four weeks in the control culture solution. Total RNA was extracted from the shoots and roots of individual plants. Expression levels were analyzed by qRT‐PCR. *Ubiquitin* and *Actin* were used as the internal standards. Expression level is shown as a value relative to that in WT plants. Values are expressed as means ± SE (*n* = 3). An asterisk (*) shows a significant difference from WT plants (*p *<* *0.05, Dunnett test)

To examine the physiological functions of OsPCS1 and OsPCS2 in vivo, we generated transgenic plants of RNAi targeting each *OsPCS* gene. Figure 4 shows the efficiency and specificity of knockdown in T_2_ plants of independent lines. In the shoots, the expression of the target gene was suppressed to 7%–14% of that in the WT in *OsPCS1* RNAi plants and 8%–18% in *OsPCS2* RNAi plants (Figure 4a). The expression of another nontarget *OsPCS* was suppressed to 60%–80% in *OsPCS2* RNAi plants and 40%–60% in *OsPCS1* RNAi plants, whereas it was significant only in a line, *PCS1* RNAi‐4 (Figure 4a). In the roots, the target gene expression was suppressed to 22%–35% of that in the WT in *OsPCS1* RNAi plants and 8%–30% in *OsPCS2* RNAi plants, but the expression level of another nontarget *OsPCS* was not decreased (Figure 4b). The difference in the levels of nontarget suppression between the shoots and the roots may be caused by the difference in the endogenous expression levels of *OsPCS* genes between the two organs. Nontarget suppression was negligible at least in the roots. In addition, the suppression of one *OsPCS* gene did not cause the upregulation of another *OsPCS* gene.

To consider Cd and As(III) stress conditions, we grew WT plants under 3, 10, and 30 μmol/L Cd and 5, 20, and 50 μmol/L As(III) for 10 days. Shoot growth of WT plants was significantly inhibited under Cd or As(III) stress in a dose‐dependent manner (Figure S6). On the other hand, root elongation was inhibited under 50 μmol/L As(III) and promoted under 3–10 μmol/L Cd and 5 μmol/L As(III) (Figure S6). Similar enhancement of root growth by low Cd was reported in miscanthus (Arduini, Masoni, Mariotti, & Ercoli, 2004). Increased demand for sulfur under moderate Cd or As stress may contribute to the promoted root elongation. The growth inhibition effect was mild in 3 μmol/L Cd and 5 μmol/L As(III) conditions but it was too severe in 30 and 50 μmol/L concentrations of Cd and As(III), respectively. Thus, we used 10 μmol/L Cd and 20 μmol/L As(III) to investigate sensitivity in the RNAi plants.

**Figure 5:**
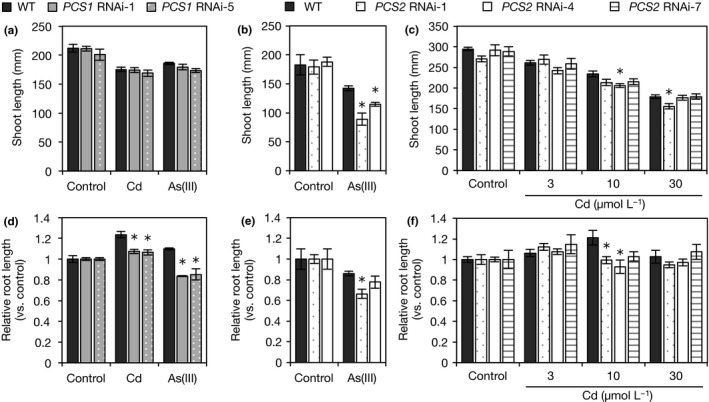
Growth of OsPCS RNAi plants under Cd or As(III) stress. (a, d) Shoot and root lengths of *OsPCS1* RNAi plants under Cd or As(III) stress. WT and *OsPCS1* RNAi plants were hydroponically grown in a culture solution containing 10 μmol/L Cd or 20 μmol/L As(III) and the control culture solution for 10 days. Root length is shown as a value relative to that in the control. Values are expressed as means ± SE (WT:* n* = 5, *PCS1* RNAi‐1: *n* = 7, *PCS1* RNAi‐5: *n* = 5). (b, e) Shoot and root lengths of *OsPCS2* RNAi plants under As(III) stress. WT and *OsPCS2* RNAi plants were hydroponically grown in a culture solution containing 20 μmol/L As(III) and the control culture solution for 10 days. Root length is shown as a value relative to that in the control. Values are expressed as means ± SE (*n* = 4). (c, f) Shoot and root lengths of *OsPCS2* RNAi plants under Cd stress. The 7‐day‐old seedlings of WT and *OsPCS2* RNAi plants were transferred to a culture solution containing 0, 3, 10, or 30 μmol/L Cd and grown for 7 days. Values are expressed as means ± SE (WT,*PCS2* RNAi‐1: *n* = 5, *PCS2* RNAi‐4, ‐7: *n* = 4). An asterisk (*) shows a significant difference from WT plants (*p *<* *0.05, Dunnett test)

Knockdown of *OsPCS1* had little effect on the sensitivity to Cd and As in rice seedlings. Shoot lengths under control conditions were similar between WT and *OsPCS1* RNAi plants, and the inhibition of shoot elongation under Cd or As(III) stress was also comparable (Figure 5a). The root growth of RNAi plants was slightly suppressed under both Cd and As(III) stress compared with that in WT plants (Figure 5d). This indicates that OsPCS1 may be involved in Cd and As tolerance to some extent, although it seems to make only a small contribution.

Knockdown of *OsPCS2* increased sensitivity to As but had a small effect on Cd tolerance in rice seedlings. Shoot lengths under control conditions were similar between WT and *OsPCS2* RNAi plants, but shoot elongation under As(III) stress was more severely inhibited in RNAi plants (Figure 5b). Reduction rates of shoot length by As(III) stress were 22% in WT plants and 39%–50% in *OsPCS2* RNAi plants. The root growth of RNAi plants under As(III) stress was also more suppressed than that of the WT (Figure 5e). In contrast, the inhibition of shoot elongation under 3–30 μmol/L Cd stress was similar between WT and *OsPCS2* RNAi plants (Figure 5c). The shoot length of one of three *OsPCS2* RNAi lines was slightly shorter than that of WT plants under 10 or 30 μmol/L Cd stress conditions; however, the other two lines did not show significant differences. The root growth of RNAi plants was significantly suppressed compared with that of WT plants only under the 10 μmol/L Cd condition (Figure 5f). This suggests that OsPCS2 plays an important role in As tolerance of rice plants but also indicates that the contribution of OsPCS to Cd tolerance is smaller than that to As tolerance.

### 3.5 PC synthesis in OsPCS RNAi plants

Syntheses of thiol peptides in the roots of RNAi plants under 10 μmol/L Cd or 20 μmol/L As(III) stress were analyzed. There was no significant difference between the thiol peptide concentrations of *OsPCS1* RNAi plants and those of WT plants (Tables 1 and 2). When *OsPCS1* RNAi plants were grown under As(III) stress, the PC_2_ concentration was slightly elevated, and PC_3, 4_ concentrations were slightly reduced compared with their concentrations in WT plants (Table 2); however, these differences were not significant. This indicates that the contribution of OsPCS1 to the overall production of PCs is small.

**Table 1:** Concentrations of thiol peptides in the roots of OsPCS RNAi plants grown under Cd stress

10 μmol/L Cd	Thiol peptides (nmol/g DW)							
Root	GSH	PC_2_	PC_3_	PC_4_	hmGSH	hmPC_2_	γECE	γEC
WT	1,000 ± 180	50.0 ± 7.9	35.6 ± 8.1	7.12 ± 1.03	1,880 ± 220	221 ± 41	299 ± 34	443 ± 42
*PCS1* RNAi‐1	969 ± 248	46.3 ± 10.2	35.3 ± 0.8	8.98 ± 1.43	1,780 ± 100	233 ± 97	276 ± 74	429 ± 23
‐4	1,230 ± 400	37.3 ± 10.9	32.5 ± 5.2	8.87 ± 4.79	2,170 ± 230	243 ± 116	302 ± 57	537 ± 40
‐5	1,170 ± 290	56.2 ± 27.6	45.5 ± 12.6	10.2 ± 3.7	2,250 ± 80	257 ± 134	302 ± 75	515 ± 33
*PCS2* RNAi‐1	1,290 ± 40	9.73* ± 9.73	n.d.*	n.d.*	1,190 ± 190	23.3* ± 15.6	16.3* ± 16.3	91.4* ± 31.1
‐4	1,510 ± 210	5.73* ± 1.45	n.d.*	n.d.*	1,080* ± 200	11.3* ± 1.6	16.5* ± 13.8	65.6* ± 10.2
‐7	1,830 ± 260	35.4 ± 14.0	2.23* ± 1.82	n.d.*	1,300 ± 400	66.3* ± 25.2	10.8* ± 1.0	95.2* ± 21.1

The 7‐day‐old seedlings of RNAi plants and wild‐type (WT) (Nipponbare) plants were transferred to a culture solution supplemented with 10 μmol/L CdSO_4_. Three plants were bulked and harvested, and the concentration of thiol peptides in the roots was determined 8 days after transfer. Values are expressed as means ± *SE* from three independent experiments. An asterisk (*) shows a significant difference from WT plants (*p *<* *0.05, Dunnett test).

**Table 2:** Concentrations of thiol peptides in the roots of OsPCS RNAi plants grown under As(III) stress

20 μmol/L As(III)	Thiol peptides (nmol/g DW)							
Root	GSH	PC_2_	PC_3_	PC_4_	hmGSH	hmPC_2_	γECE	γEC
WT	1,250 ± 210	1,820 ± 340	553 ± 155	57.3 ± 15.6	2,630 ± 250	3,000 ± 180	2,020 ± 260	2,800 ± 230
*PCS1* RNAi‐1	1,710 ± 270	2,230 ± 350	333 ± 87	28.9 ± 8.8	2,530 ± 140	2,600 ± 200	2,100 ± 160	2,860 ± 280
‐4	1,800 ± 440	2,350 ± 310	364 ± 111	37.2 ± 8.2	2,720 ± 110	2,870 ± 110	2,260 ± 280	3,340 ± 510
‐5	1,880 ± 450	2,450 ± 360	423 ± 110	38.8 ± 7.9	2,370 ± 320	2,650 ± 230	2,200 ± 320	3,250 ± 490
*PCS2* RNAi‐1	6,680* ± 1590	1,110 ± 160	182* ± 58	17.1* ± 5.6	2,800 ± 330	861* ± 99	1,280 ± 80	3,780 ± 750
‐4	6,750* ± 1200	1,310 ± 280	214* ± 77	32.8 ± 12.9	2,960 ± 310	922* ± 170	1,330 ± 250	4,190 ± 470
‐5	4,460* ± 430	1,650 ± 80	273 ± 61	35.4 ± 7.8	2,240 ± 170	1,280* ± 230	1,430 ± 150	3,580 ± 110

The 7‐day‐old seedlings of RNAi plants and wild‐type (WT) (Nipponbare) plants were transferred to a culture solution supplemented with 20 μmol/L NaAsO_2_. Three plants were bulked and harvested, and the concentration of thiol peptides in the roots was determined 8 days after transfer. Values are expressed as means ± *SE* from three independent experiments. An asterisk (*) shows a significant difference from WT plants (*p *<* *0.05, Dunnett test).

In contrast, *OsPCS2* RNAi plants showed a significant reduction in PC concentration under both Cd and As(III) stresses compared with WT plants. When grown with Cd, the concentrations of PC_2_ and hmPC_2_ were severely decreased in the two RNAi lines, *PCS2* RNAi‐1 and *PCS2* RNAi‐4, where *OsPCS2* expression was suppressed to 8% of the WT. In these RNAi plants, PC_3_ and PC_4_ were not detected (Table 1). Furthermore, the concentrations of GSH analogs, such as hmGSH, γECE, and γEC, were also reduced in *OsPCS2* RNAi plants grown under Cd stress. The concentration of GSH, a precursor of PCs, was slightly increased in RNAi plants compared with that in the WT; however, this difference was not significant. When grown under As(III) stress, the syntheses of PC_2–4_ and hmPC_2_ were diminished in *OsPCS2* RNAi plants (Table 2), although the reduction rate was not as severe as that under Cd stress. The GSH concentration was several‐fold higher in *OsPCS2* RNAi plants than that in WT plants under As(III) stress. This suggests the involvement of OsPCS2 in PC synthesis in response to not only Cd but also As(III) stress in rice.

### 3.6 Cd and As accumulation in OsPCS RNAi plants

**Figure 6:**
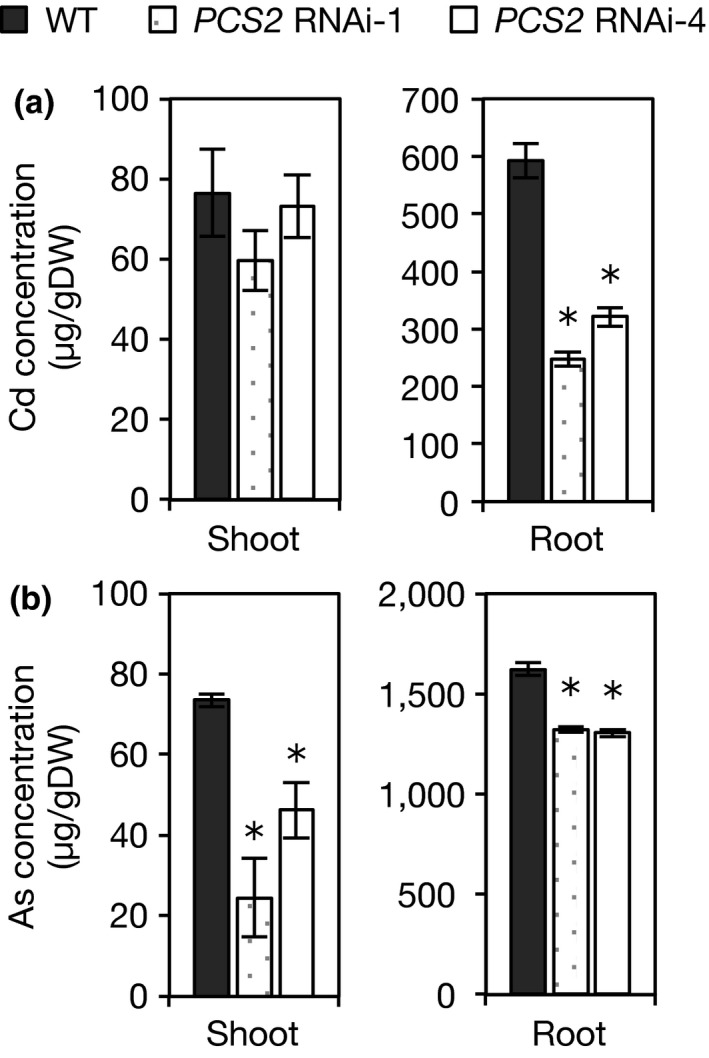
Cd and As concentrations in OsPCS2 RNAi plants. (a) Cd concentration in shoots (left) and roots (right). (b) As concentration in shoots (left) and roots (right). WT and *OsPCS2* RNAi plants were hydroponically grown in a culture solution containing 10 μmol/L Cd or 20 μmol/L As(III) for 10 days. Each plant was separated into shoots and roots and oven‐dried. After digestion with HNO_3_ and HClO_4_, Cd concentration was analyzed by atomic absorption spectroscopy and As concentration was analyzed by inductively coupled plasma atomic emission spectroscopy. Values are expressed as means ± *S*E (*n* = 4). An asterisk (*) shows a significant difference from WT plants (*p *<* *0.05, Dunnett test)

The accumulation of Cd and As did not differ between WT and *OsPCS1* RNAi plants; however, a reduction was observed in *OsPCS2* RNAi plants. The Cd concentration in the shoots and the As concentration in both the shoots and the roots of *OsPCS1* RNAi plants did not differ from their concentrations in WT plants (Figure S7). Although Cd concentration in the roots of *OsPCS1* RNAi plants was significantly lower or higher than that in the roots of WT plants, it was not consistent between the two RNAi lines. On the other hand, the concentrations of Cd and As in the roots of *OsPCS2* RNAi plants were significantly lower than their levels in WT plants (Figure 6). The As concentration in the shoots of *OsPCS2* RNAi plants was also significantly lower than that in the shoot of WT plants. However, the Cd concentration in their shoots did not differ from that in the shoots of WT plants.

### 3.7 Effect of GSH synthesis inhibition

We hypothesized that, instead of PCs, GSH might contribute to Cd tolerance in rice. To test this hypothesis, we investigated the effect of inhibiting GSH synthesis.

**Figure 7:**
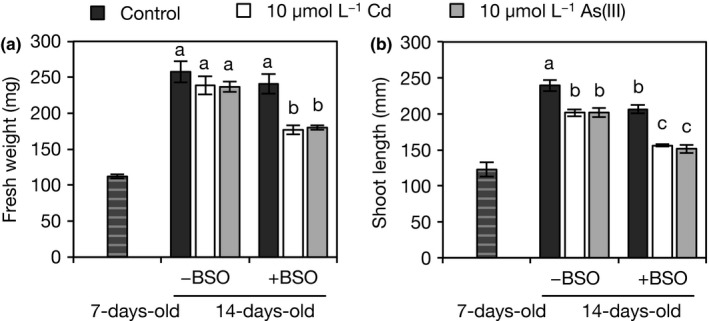
Effect of a GSH synthesis inhibitor on Cd and As sensitivity. (a) Fresh weights and (b) shoot lengths of rice seedlings. The 7‐day‐old seedlings of Nipponbare were transferred to a culture solution supplemented or not with toxic metals [10 μmol/L Cd or 10 μmol/L As(III)] either alone or in combination with 0.5 mmol/L BSO and grown for 7 days. Fresh weight and shoot length were measured before and after the treatment. Values are expressed as means ± *SE* (*n* = 5). Different letters show a significant difference (*p *<* *0.05, Tukey's test)

BSO is a specific inhibitor of GSH biosynthesis that causes the depletion of cellular GSH levels. BSO is a transition state analog and it tightly binds to the active site of γEC synthetase; the enzyme catalyzes the first step reaction of GSH synthesis (Griffith, 1982). In our analysis, WT seedlings were grown under Cd or As(III) stress with or without BSO. In the absence of BSO, the fresh weight of the whole plant was not reduced by Cd or As(III) stress. However, the addition of BSO resulted in a significant reduction in the fresh weights and shoot lengths of rice seedlings under both Cd and As(III) stress conditions (Figure 7). Synthesis of thiol peptides in response to Cd and As(III) was almost completely abolished by BSO treatment (Table S2). This suggests that GSH synthesis is crucial for Cd and As tolerance in rice plants.

**Figure 8:**
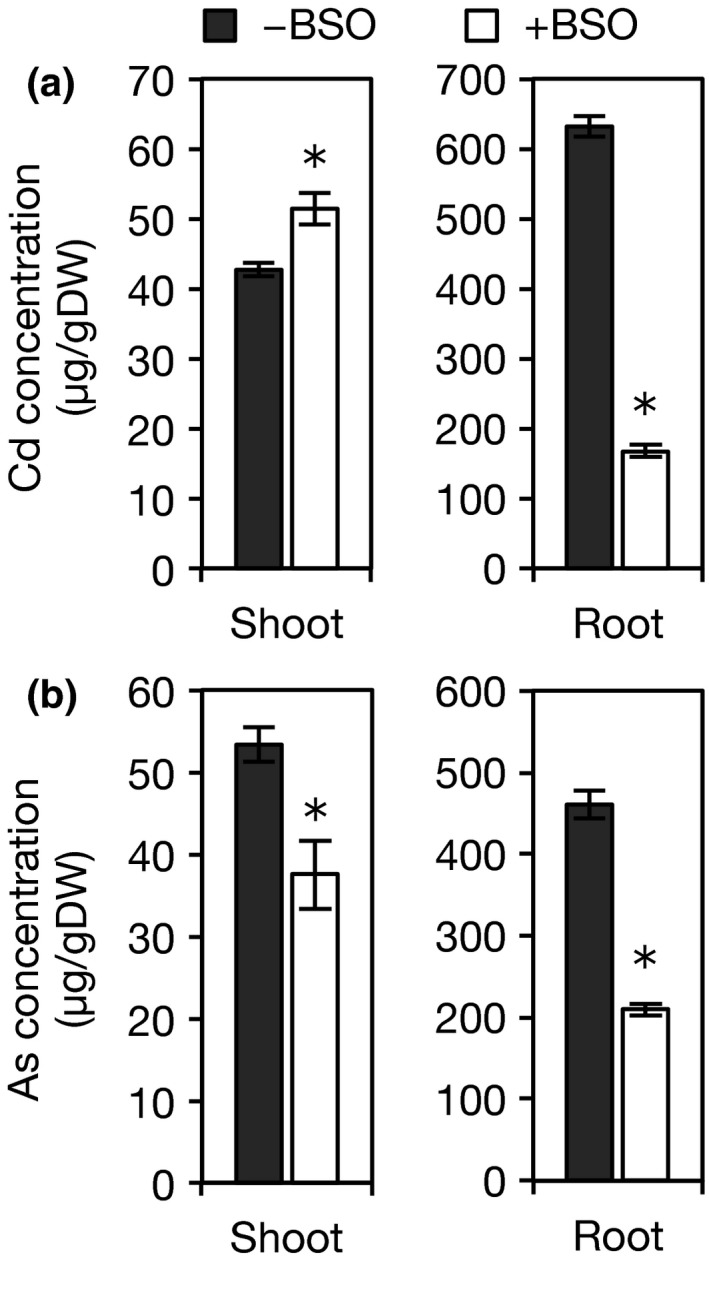
Effect of a GSH synthesis inhibitor on Cd and As concentrations. (a) Cd concentration in shoots (left) and roots (right). (b) As concentration in shoots (left) and roots (right). Seven‐day‐old seedlings of Nipponbare were transferred to a culture solution supplemented with toxic metals [10 μmol/L Cd or 10 μmol/L As(III)] either alone or in combination with 0.5 mmol/L BSO and grown for 7 days. Each plant was separated into shoots and roots and oven‐dried. After digestion with HNO_3_ and HClO_4_, Cd concentration was analyzed by atomic absorption spectroscopy, and As concentration was analyzed by inductively coupled plasma atomic emission spectroscopy. Values are expressed as means ± *SE* (*n* = 5). An asterisk (*) shows a significant difference (*p *<* *0.05, Dunnett test)

The effect on Cd and As accumulation in rice seedlings was also investigated. The addition of BSO significantly reduced the concentration of As in both shoots and roots (Figure 8b). Although the addition of BSO also caused a remarkable reduction in the Cd concentration of the roots, it caused an elevation in the Cd concentration of the shoots (Figure 8a). This indicates that GSH suppresses the translocation of Cd from the roots to the shoots.

## 4 DISCUSSION

### 4.1 Physiological functions of OsPCS1 and OsPCS2

On the basis of information provided by bioinformatic tools or public databases, it is now clear that *PCS*‐like genes are ubiquitous in higher plants and there are at least two *PCS* genes in most plant species (Clemens, 2006). As *PCS*‐like genes in rice, *Os05g0415200* (*OsPCS1*) and *Os06g0102300* (*OsPCS2*) were found in the RAP‐DB. We designated *Os05g0415200* as *OsPCS1* and *Os06g0102300* as *OsPCS2* according to a guideline for gene nomenclature in rice that suggested that genes be numbered in order of discovery (McCouch, 2008) because the symbol *OsPCS1* had been applied to a transcript (AF439787) arising from the locus *Os05g0415200* in an earlier report (Li et al., 2007). However, the numbering of *OsPCS* genes did not coincide with among three recently published reports (Das et al., 2017; Hayashi et al., 2017; Uraguchi et al., 2017). Our designation is the same as in the reports by Das et al. (2017) and Hayashi et al. (2017).

In the present study, we investigated the physiological contributions of *OsPCS1* and *OsPCS2* to Cd and As tolerance using transgenic plants of RNAi‐mediated knockdown. The knockdown of *OsPCS1* had no significant effect on the concentration of thiol peptides under Cd or As stress (Tables 1 and 2). The expression of a nontarget gene, *OsPCS2* in *OsPCS1* RNAi plants, and vice versa, did not increase or decrease in the RNAi roots (Figure 4b). These results suggest that OsPCS1 makes little contribution to overall PC synthesis at the seedling stage. In addition, *OsPCS1* RNAi plants did not show a clear effect on Cd and As tolerance, which is consistent with a recent report showing that a loss‐of‐function mutant of *OsPCS1* (*has2*) was not sensitive to Cd and As (Hayashi et al., 2017). Regarding the physiological function of OsPCS1, Hayashi et al. (2017) suggested that OsPCS1 was crucial for reducing As levels in rice grains by trapping As in the upper node. This does not conflict with our findings obtained here; *OsPCS1* expression in the shoots was higher than that in the roots (Figure S4) and the activation of OsPCS1 in vitro by As(III) was stronger than by Cd (Figure 3c). OsPCS1 has a minor role in PC synthesis at the seedling stage, but it may perform tissue‐specific or growth‐stage‐specific functions in rice plants.

In contrast to *OsPCS1*, knockdown of *OsPCS2* significantly decreased the concentration of PCs in the roots (Tables 1 and 2) and affected the As sensitivity of rice plants. Considering that nontarget *OsPCS1* has been expressed in *OsPCS2* RNAi plants, we suggest that OsPCS2 is a major PCS isozyme that controls PC synthesis in response to toxic heavy metals/metalloids. This is supported by the result that the *OsPCS2* expression level was higher than *OsPCS1* in shoots and roots (Figure 2, Figure S4). On the other hand, there is the contrasting activator preference between in vivo PC synthesis and in vitro OsPCS2 assay. As(III) stress strongly induced PC synthesis in the rice roots (Figure 1), but Cd^2+^ highly activated in vitro OsPCS2‐catalyzed PC formation (Figure 3). This suggests that PC synthesis in rice plants is regulated not just through activation by heavy metals/metalloids. As both OsPCS1 and OsPCS2 are localized in the cytosol (Hayashi et al., 2017), the cytosolic concentration of GSH, a substrate, or Cd^2+^, an activator, may be insufficient for PC formation in rice cells under Cd stress due to PC‐independent Cd sequestration.

We found three alternatively spliced variants of *OsPCS2* by sequence analysis, and the longest variant *OsPCS2*#1 was the dominantly expressed transcript in both the shoots and the roots under the control and Cd or As(III) stress conditions (Figure 2c, Figure S4b). In a recent study, Uraguchi et al. (2017) also isolated additional *OsPCS2* variants, which were designated as *OsPCS1* variants in their article, having a different transcription start site, and they reported that the longest *OsPCS2* variant, *OsPCS1full*, showed the highest abundance among *OsPCS* transcripts. In contrast, Das et al. (2017) reported that the longest *OsPCS2* variant was abundant in the shoots but not in the roots of *indica* rice (cv. IR64) under Cd stress, even though the variant is identical to our *OsPCS2*#1. They used a high concentration of Cd (100 μmol/L) for the expression analysis (Das et al., 2017), whereas we used a lower concentration (10 μmol/L Cd) in the present study. Such a difference in Cd level, and also the difference in cultivar (*indica* IR64 or *japonica* Nipponbare), could have caused the different expression patterns of *OsPCS2* variants.

### 4.2 Different contributions of PC synthesis to As and Cd tolerance in rice

It is known that PCS has a crucial role in heavy metal tolerance in higher plants, as revealed by data showing that *Arabidopsis* PC‐deficient mutant, *cad1‐3* which had a mutation in *AtPCS1*, was highly sensitive to Cd and As (Ha et al., 1999; Howden et al., 1995). In the present study, *OsPCS2* RNAi rice plants contained less PCs under As(III) stress (Table 2), and they were more sensitive to As than were WT plants (Figure 5b). This suggests that PC synthesis is crucial for As tolerance in rice. In plant cells, As(III) is complexed with PCs and sequestered into vacuoles (Song et al., 2010). Previously, Song et al. (2014) reported that the knockout of *OsABCC1*, a rice ABC transporter gene involved in the vacuolar sequestration of PC‐As complex, resulted in significantly decreased As tolerance in rice. Our findings and the previous report suggest that both steps, PC synthesis and vacuolar sequestration of the PC‐As complex, are important for As detoxification in rice cells. In addition, PC synthesis in the rice roots was more strongly induced by As(III) exposure than by Cd exposure (Figure 1). Such a preference in activators may be attributable to the bioavailability of As and Cd in the habitat. Under anaerobic soil conditions such as a paddy field, Cd solubility is low but As is present in a highly soluble form As(III); thus, Cd uptake by rice is suppressed but As(III) uptake is enhanced in a flooded soil environment (Arao et al., 2009). Furthermore, rice takes up As(III) more easily than other plants do because of its efficient transport pathway (Su et al., 2010). Accommodation to anaerobic soil conditions and As exposure may result in the strong induction of PC synthesis by As stress in rice.

Surprisingly, we found that knockdown of *OsPCS2* had a small effect on Cd tolerance of rice seedlings (Figure 5c) even though PC synthesis was remarkably suppressed (Table 1). From this result, we suppose that PC synthesis in rice may make a small contribution to Cd tolerance compared with its crucial role for As tolerance. Similar phenotypes were observed for other mutants involved in PC synthesis and transport. Recently, it was reported that the T‐DNA mutant of *OsPCS2* showed a weak phenotype under Cd stress, in contrast to a clear decrease in As tolerance (Uraguchi et al., 2017). In addition, the knockout of *OsABCC1* did not affect Cd toxicity in rice (Song et al., 2014), in contrast to *Arabidopsis atabcc1 atabcc2* mutant showing high sensitivity to As and Cd (Park et al., 2012; Song et al., 2010). Such our findings and findings from previous reports may indicate that Cd tolerance of rice can be mediated through PC‐independent mechanisms.

Different contribution of PCs to As and Cd tolerance may be attributable to the pathway and chemical form of them in vacuolar sequestration. In yeast heterologous expression analyses, OsABCC1 enhances PC‐dependent As resistance but does not affect Cd tolerance (Song et al., 2014), suggesting that OsABCC1 has high selectivity for the PC‐As complex but a low affinity for the PC‐Cd complex. Therefore, the vacuolar sequestration of Cd may be mediated mostly via another pathway in rice. One possible pathway is OsHMA3‐mediated Cd^2+^ sequestration into a vacuole. OsHMA3 is a rice tonoplast‐localized P_1B_‐ATPase limiting the root‐to‐shoot Cd translocation via the sequestration of Cd^2+^ into root vacuoles (Miyadate et al., 2011; Ueno et al., 2010). *Arabidopsis* also has a homolog AtHMA3 (Morel et al., 2009), whereas it is nonfunctional in the Columbia ecotype (background of *cad1‐3* and *atabcc1 atabcc2* mutants) owing to a mutation (Hussain et al., 2004). Another possible pathway is GSH *S*‐conjugate transport like in budding yeast (*Saccharomyces cerevisiae*), in which the yeast cadmium factor 1 (YCF1) mediates vacuolar accumulation of glutathione‐Cd (GS‐Cd) complexes and confers Cd resistance (Li, Szczypka, Lu, Thiele, & Rea, 1996; Li et al., 1997). Although plant vacuolar GS‐Cd transporters have not been isolated, *YCF1*‐expressing *Arabidopsis* plants show increased GS‐Cd uptake into intact vacuoles and enhanced Cd resistance (Song et al., 2003). This hypothesis can be supported by the growth experiment with BSO in the present study. It revealed that the inhibition of GSH biosynthesis significantly enhanced Cd sensitivity in rice seedlings (Figure 7), suggesting that GSH plays a crucial role in Cd tolerance, not only as a substrate of PC synthesis. We suppose that GSH itself may bind to Cd and mitigate its toxicity. In addition, the role of GSH as a scavenger of reactive oxygen species produced by Cd stress in plants (Jozefczak, Remans, Vangronsveld, & Cuypers, 2012) may contribute to the alleviation of Cd toxicity.

In *OsPCS2* RNAi plants under As(III) stress, GSH accumulated at a several‐fold higher level than it did in WT plants (Table 2). Too high a concentration of GSH in plants could paradoxically enhance sensitivity to oxidative stress (Creissen et al., 1999), whereas we did not observe oxidative damage such as chlorosis or necrosis in *OsPCS2* RNAi plants. As the inhibition of GSH biosynthesis and subsequent decrease in PCs also resulted in high As sensitivity of rice seedlings (Figure 7), the enhanced As sensitivity of *OsPCS2* RNAi plants is most likely due to a decrease in PCs rather than a great increase in GSH. In contrast, under Cd stress, the GSH concentration in *OsPCS2* RNAi plants was slightly increased but it did not differ significantly from that in WT plants (Table 1). This might be because the concentrations of PCs even in WT plants were much lower than that of GSH under Cd stress. In addition, *OsPCS2* RNAi plants might not undergo Cd stress as severely as As(III) stress.

### 4.3 Accumulation and translocation of As and Cd in rice plants

In our transgenic plant experiment, the silencing of *OsPCS2* caused a decrease in As and Cd concentrations in the roots (Figure 6), suggesting that OsPCS2 is involved in As and Cd accumulation in the rice roots. This is consistent with previous reports describing that *Arabidopsis* PC‐deficient mutant *cad1‐3* showed less accumulation of As(III) and Cd in the roots (Liu et al., 2010; Wong & Cobbett, 2009). In *Arabidopsis*, complexation of As with PCs in the roots decreases As(III) efflux and root‐to‐shoot translocation (Liu et al., 2010). Interestingly, we found that the suppression of PC synthesis did not enhance As translocation from roots to shoots in rice, based on the results that the As concentration was reduced also in the shoots of *OsPCS2* RNAi plants and BSO‐treated WT plants. This indicates that nonbound As(III) in the rice roots may be efficiently discharged to the external medium via an aquaporin channel Lsi1 (OsNIP2;1) and other unidentified As(III) efflux pathways (Zhao et al., 2010) rather than loaded into the xylem. Another possibility is that PCs may also be involved in the translocation of As from the roots to the shoots. Although in the rice roots most As is loaded into the xylem in its ionic form via the silicic acid transporter Lsi2 (Ma et al., 2008), some of the PC‐As complex may be transferred to the shoots via the xylem or phloem transport pathway in rice.

On the other hand, the shoot Cd concentration was not affected by *OsPCS2* knockdown, but it was increased by the inhibition of GSH biosynthesis. As the syntheses of PCs and GSH‐like peptides were suppressed in *OsPCS2* RNAi roots but the GSH concentration was comparable or slightly higher than in the WT (Table 1), it is suggested that GSH may have roles in the retention of Cd in rice roots and the suppression of root‐to‐shoot Cd translocation.

In *OsPCS1* RNAi plants, the concentrations of Cd and As in the shoots and roots were not affected (Figure S7), even though the nontarget *OsPCS2* expression in the shoots was suppressed to <60% of that in the WT (Figure 4a). Such nontarget suppression was not observed in the roots (Figure 4b), and also the synthesis of thiol peptides in the roots was not significantly affected in *OsPCS1* RNAi plants (Tables 1 and 2). This indicates that PCS2 activity in rice roots regulates Cd and As accumulation in the roots and As translocation to the shoots.

### 4.4 OsPCS2 is involved in the synthesis of GSH‐like peptides

Some plant species produce GSH‐like peptides including hmGSH in Poaceae (Klapheck, Chrost, Starke, & Zimmermann, 1992) and γECE in maize (*Zea mays*) (Meuwly, Thibault, & Rauser, 1993), in which Ser or Glu replaces Gly in the tripeptide. Although the biosynthetic pathways of hmGSH and γECE remain unidentified, it has been estimated that the peptides are synthesized through ATP‐dependent ligation from γEC and Ser or Glu, like in GSH synthesis, or through postsynthetic modifications of GSH, like in the transpeptidation of PC synthesis (Galant, Preuss, Cameron, & Jez, 2011; Skipsey, Davis, & Edwards, 2005). In the present study, accumulations of hmGSH, γECE and γEC were observed in the rice roots under Cd stress (Figure 1), but interestingly we found that *OsPCS2* knockdown decreased the concentrations of γECE and γEC severely and that of hmGSH to half the level of WT plants (Table 1). OsPCS2 in vitro did not synthesize hmGSH or γECE from GSH and Ser or Glu; however, OsPCS2‐catalyzed γEC production from GSH was detected (Figure 3b). These results suggest that OsPCS2 is involved in the synthesis of hmGSH and γECE under Cd stress, but it makes an indirect contribution. In plants, γEC is synthesized exclusively in the plastid (Wachter, Wolf, Steininger, Bogs, & Rausch, 2005) and it is probably rapidly converted into GSH. The resultant GSH or γEC in the plastid is exported to the cytosol, which is required for GSH homeostasis in *Arabidopsis* and rice (Maughan et al., 2010; Yang et al., 2016). On the other hand, PCS can catabolize GSH conjugates into γEC conjugates in the cytosol and enzyme activity depends on the presence of Cd^2+^ (Beck, Lendzian, Oven, Christmann, & Grill, 2003; Blum, Meyer, Wünschmann, Lendzian, & Grill, 2010; Grzam, Tennstedt, Clemens, Hell, & Meyer, 2006). Taking these results and previous reports into consideration, OsPCS2 may catalyze the conversion of GSH into γEC in the cytosol under Cd stress, and the resultant γEC may be used as a substrate in a subsequent hmGSH or γECE synthetic reaction catalyzed by unknown enzymes.

## 5 CONCLUSION

In conclusion, the two rice PC synthase isozymes, OsPCS1 and OsPCS2, have different enzymatic properties in the activation by Cd and As. Of them, OsPCS2 is a major isozyme controlling PC synthesis in response to Cd and As. PC synthesis is crucial for As tolerance in rice, but it may make a smaller contribution to Cd tolerance than that to As tolerance. In rice, GSH plays a crucial role in coping with Cd toxicity not only as a substrate of PC synthesis. Further investigations about the function of GSH under Cd stress in rice cells should make a substantial contribution to our understanding of Cd detoxification mechanisms in rice.

## AUTHOR CONTRIBUTIONS

S.Y. performed most of the experiments and drafted the article with contributions from all authors; Y.U. conducted the enzyme experiment; A.M. analyzed the synthesis of thiol peptides in the wild‐type plants in response to heavy metals; and K.O. and T.M. designed the research and supervised the experiments.

## ACCESSION NUMBERS

The nucleotide sequence reported in this paper has been submitted to the DNA Data Bank of Japan with accession numbers LC192427, LC192428, LC192429, LC192430, and LC192431.
