# Supplementary material for: Rice phytochelatin synthases OsPCS1 and OsPCS2 make different contributions to cadmium and arsenic tolerance
Source: Plant Direct. 2018 Jan 9;2(1):e00034. doi: 10.1002/pld3.34 (PMC6508543; doi:10.1002/pld3.34)
Supplement: Supplementary file 1 [file PLD3-2-e00034-s001.pdf]

(A) *OsPCS1*

```
Genome     161>TTTTGCCGCAATGGCGTCTAAACCAAGCAGCCGAGCGGAAAGCAACCAGGCGGCGGCGGCGGTGCCGTCGCTGTACGGGC>240
CB646798     1>~~~~~~~~~~~ATGGCGTCTAAACCAAGCAGCCGAGCGGAAAGCAACCAGGCGGCGGCGGCGGTGCCGTCGCTGTACGGGC>70
OsPCS1#1   161>TTTTGCCGCAATGGCGTCTAAACCAAGCAGCCGAGCGGAAAGCAACCAGGCGGCGGCGGCGGTGCCGTCGCTGTACGGGC>240
OsPCS1#2     1>~~~~~~~~~~~ATGGCGTCTAAACCAAGCAGCCGAGCGGAAAGCAACCAGGCGGCGGCGGCGGTGCCGTCGCTGTACGGGC>70
OsPCS1#3     1>~~~~~~~~~~~ATGGCGTCTAAACCAAGCAGCCGAGCGGAAAGCAACCAGGCGGCGGCGGCGGTGCCGTCGCTGTACGGGC>70
OsPCS1#4     1>~~~~~~~~~~~ATGGCGTCTAAACCAAGCAGCCGAGCGGAAAGCAACCAGGCGGCGGCGGCGGTGCCGTCGCTGTACGGGC>70
                        *         *         *         *         *         *         *         *
                        *         *         *         *         *         *         *         *

Genome     641>GGATTGCCATCTCGTCGCCTCCTACCACCGGAAGCTTCTCGGCCAGGTTGCTGCCTGCTCGTCCGCACCTCTCTCGGAAC>720
CB646798   471>GGATTGCCATCTCGTCGCCTCCTACCACCGGAAGCTTCTCGGCCAG---------------------------------->516
OsPCS1#1   641>GGATTGCCATCTCGTCGCCTCCTACCACCGGAAGCTTCTCGGCCAG---------------------------------->686
OsPCS1#2   471>GGATTGCCATCTCGTCGCCTCCTACCACCGGAAGCTTCTCGGCCAG---------------------------------->516
OsPCS1#3   471>GGATTGCCATCTCGTCGCCTCCTACCACCGGAAGCTTCTCGGCCAG---------------------------------->516
OsPCS1#4   471>GGATTGCCATCTCGTCGCCTCCTACCACCGGAAGCTTCTCGGCCAG---------------------------------->516
                        *         *         *         *         *         *         *         *

Genome     721>TCGAATTCGACGATTTCTGTTTGCTGTTCGTTCTGAACGAATGATGTTTGTTTGGATTCGATGCAGACTGGAACAGGGCA>800
CB646798   517>------------------------------------------------------------------ACTGGAACAGGGCA>530
OsPCS1#1   687>------------------------------------------------------------------ACTGGAACAGGGCA>700
OsPCS1#2   517>------------------------------------------------------------------ACTGGAACAGGGCA>530
OsPCS1#3   517>------------------------------------------------------------------ACTGGAACAGGGCA>530
OsPCS1#4   517>------------------------------------------------------------------ACTGGAACAGGGCA>530
                        *         *         *         *         *         *         *         *
                        *         *         *         *         *         *         *         *

Genome     881>ATTGGATTCCGCTGCCGCTTCTTTGGGAAGCCATGAACACGATTGATGAAGCAACTGGGCTTCTCAGGGGGTATATATTC>960
CB646798   611>ATTGGATTCCGCTGCCGCTTCTTTGGGAAGCCATGAACACGATTGATGAAGCAACTGGGCTTCTCAGGGG---------->680
OsPCS1#1   781>ATTGGATTCCGCTGCCGCTTCTTTGGGAAGCCATGAACACGATTGATGAAGCAACTGGGCTTCTCAGGGG---------->850
OsPCS1#2   611>ATTGGATTCCGCTGCCGCTTCTTTGGGAAGCCATGAACACGATTGATGAAGCAACTGGGCTTCTCAGGGG---------->680
OsPCS1#3   611>ATTGGATTCCGCTGCCGCTTCTTTGGGAAGCCATGAACACGATTGATGAAGCAACTGGGCTTCTCAGGGG---------->680
OsPCS1#4   611>ATTGGATTCCGCTGCCGCTTCTTTGGGAAGCCATGAACACGATTGATGAAGCAACTGGGCTTCTCAGGGG---------->680
                        *         *         *         *         *         *         *         *
                        *         *         *         *         *         *         *         *

Genome    1761>GTTTAACATGTCGGCATCTTCATATTTGGTAGGTTCATGCTTATCTCAAGGAATACTGAAGCTCCTTTATTGATCCGTGC>1840
CB646798   681>--------------------------------GTTCATGCTTATCTCNAGGAATACTGAAGCTCCTTTATTGATCCGTGC>728
OsPCS1#1   851>--------------------------------GTTCATGCTTATCTCAAGGAATACTGAAGCTCCTTTATTGATCCGTGC>898
OsPCS1#2   681>--------------------------------GTTCATGCTTATCTCAAGGAATACTGAAGCTCCTTTATTGATCCGTGC>728
OsPCS1#3   680>-------------------------------------------------------------------------------->680
OsPCS1#4   681>--------------------------------GTTCATGCTTATCTCAAGGAATACTGAAGCTCCTTTATTGATCCGTGC>728
                        *         *         *         *         *         *         *         *

Genome    1841>AGTGGTAAATTAACATGCCAATGCTTGGTGAATCAAATATGTAAGTGTCAAGTGTCACCCTTGATCTATTTTTTTCTCAT>1920
CB646798   729>AGTG---------------------------------------------------------------------------->732
OsPCS1#1   899>AGTG---------------------------------------------------------------------------->902
OsPCS1#2   729>AGTG---------------------------------------------------------------------------->732
OsPCS1#3   681>--TGGTAAATTAACATGCCAATGCTTGGTGAATCAAATAT---------------------------------------->718
OsPCS1#4   729>AGTGGTAAATTAACATGCCAATGCTTGGTGAATCAAATAT---------------------------------------->768
                        *         *         *         *         *         *         *         *

Genome    1921>TCCTTTCAATGCTATGCAGAATTGCAGGGATGAAAGCTGGCAAAGCATGGCAAAGTATTGCATAGAAGTTGTCCCAAATC>2000
CB646798   733>-------------------AATTGCAGGGATGAAAGCTGGCAAAGCATGGCAAAGTATTGCA~~~~~~~~~~~~~~~~~~>775
CK083842     8>---------------------------------------------------------------AGAAGTTGTCCCAAATC>24
OsPCS1#1   903>-------------------AATTGCAGGGATGAAAGCTGGCAAAGCATGGCAAAGTATTGCATAGAAGTTGTCCCAAATC>963
OsPCS1#2   733>-------------------AATTGCAGGGATGAAAGCTGGCAAAGCATGGCAAAGTATTGCATAGAAGTTGTCCCAAATC>793
OsPCS1#3   718>-------------------------------------------------------------------------------->718
OsPCS1#4   768>-------------------------------------------------------------------------------->768
                        *         *         *         *         *         *         *         *

Genome    2001>TTTTGAGGGATAACAGCGTAGACAATGTCCTAACAATTCTTTCCCGTTTAGTGAATCATCTTCCTCCCAATGCTGGAAAT>2080
CK083842    25>TTTTGAGGGATAACAGCGTAGACAATGTCCTAACAATTCTTTCCCGTTTAGTGAATCATCTTCCTCCCAATGCTGGAAAT>104
OsPCS1#1   964>TTTTGAGGGATAACAGCGTAGACAATGTCCTAACAATTCTTTCCCGTTTAGTGAATCATCTTCCTCCCAATGCTGGAAAT>1043
OsPCS1#2   794>TTTTGAGGGATAACAGCGTAGACAATGTCCTAACAATTCTTTCCCGTTTAGTGAATCATCTTCCTCCCAATGCTGGAAAT>873
OsPCS1#3   719>---------------------------------------------------TGAATCATCTTCCTCCCAATGCTGGAAAT>747
OsPCS1#4   769>---------------------------------------------------TGAATCATCTTCCTCCCAATGCTGGAAAT>797
                        *         *         *         *         *         *         *         *
                        *         *         *         *         *         *         *         *

Genome    2161>CCTGAAGGTCATTTTAATTTTCCATTTCTGTTGTTGGGTCAGTTTTGTTTCCTCTGTTCAACCAGTTAATGTTGTATGTT>2240
CK083842   185>CCTGAAG------------------------------------------------------------------------->191
OsPCS1#1  1124>CCTGAAG------------------------------------------------------------------------->1130
OsPCS1#2   954>CCTGAAG------------------------------------------------------------------------->960
OsPCS1#3   828>CCTGAAG------------------------------------------------------------------------->834
OsPCS1#4   878>CCTGAAG------------------------------------------------------------------------->884
                        *         *         *         *         *         *         *         *

Genome    2241>CGCTTGCAGGAAAAGGTCCTACAGCAAATCCGTGATACTAAGCTTTTTCAGCTGGTCCACAAACTGCAATGTTCTAAGCA>2320
CK083842   192>---------GAAAAGATCCTACAGCAAATCCGTGATACTAGGCTTTTTCAGCTGGTCCACAAACTGCAATGTTCTAAGCA>262
CB646799   794<-----------------------GCAAATCCGTGATACTAAGCTTTT-CAGCTGGTCCACAAACTGCAATGTTCTAAGCA<739
OsPCS1#1  1131>---------GAAAAGGTCCTACAGCAAATCCGTGATACTAAGCTTTTTCAGCTGGTCCACAAACTGCAATGTTCTAAGCA>1201
OsPCS1#2   961>---------GAAAAGGTCCTACAGCAAATCCGTGATACTAAGCTTTTTCAGCTGGTCCACAAACTGCAATGTTCTAAGCA>1031
OsPCS1#3   835>---------GAAAAGGTCCTACAGCAAATCCGTGATACTAAGCTTTTTCAGCTGGTCCACAAACTGCAATGTTCTAAGCA>905
OsPCS1#4   885>---------GAAAAGGTCCTACAGCAAATCCGTGATACTAAGCTTTTTCAGCTGGTCCACAAACTGCAATGTTCTAAGCA>955
                        *         *         *         *         *         *         *         *
```

```
Genome    2321>GCCCTGTTGTAGTTGCTCGTCTTTAACGGACGAAGATTCCATTTCCCAGATTGCAGCCAGTGTGTGCTGTGAAGCAACCG>2400
CK083842   263>GCCCTGTTTTAGTTGCTCGTCTTTAACGGACGAAGATTCCATTTCCCAGATTGCAGCCAGTGTGTGCTGTGAAGCAACCG>342
CB646799   738<GCCCTGTTGTAGTTGCTCGTCTTTAACGGACGAAGATTCCATTTCCCAGATTGCAGCCAGTGTGTGCTGTGAAGCAACCG<659
OsPCS1#1  1202>GCCCTGTTGTAGTTGCTCGTCTTTAACGGACGAAGATTCCATTTCCCAGATTGCAGCCAGTGTGTGCTGTGAAGCAACCG>1281
OsPCS1#2  1032>GCCCTGTTGTAGTTGCTCGTCTTTAACGGACGAAGATTCCATTTCCCAGATTGCAGCCAGTGTGTGCTGTGAAGCAACCG>1111
OsPCS1#3   906>GCCCTGTTGTAGTTGCTCGTCTTTAACGGACGAAGATTCCATTTCCCAGATTGCAGCCAGTGTGTGCTGTGAAGCAACCG>985
OsPCS1#4   956>GCCCTGTTGTAGTTGCTCGTCTTTAACGGACGAAGATTCCATTTCCCAGATTGCAGCCAGTGTGTGCTGTGAAGCAACCG>1035
                        *         *         *         *         *         *         *         *
                        *         *         *         *         *         *         *         *

Genome    2481>GAGGGGCTTAAAAATGTCATTACAGGCAAAGTGGTATCTGAAGGCAATGGACATGTTGATAAGCTTTCACCGATATCCTC>2560
CK083842   423>GAGGGGCTTAAAAATGGCATTACAGGCAAAGTGGTATCTGAAGGCAATGGACATGTTGATAAGCTTTCACCGATATCCTC>502
CB646799   578<GAGGGGCTTAAAAATGTCATTACAGGCAAAGTGGTATCTGAAGGCAATGGACATGTTGATAAGCTTTCACCGATATCCTC<499
OsPCS1#1  1362>GAGGGGCTTAAAAATGTCATTACAGGCAAAGTGGTATCTGAAGGCAATGGACATGTTGATAAGCTTTCACCGATATCCTC>1441
OsPCS1#2  1192>GAGGGGCTTAAAAATGTCATTACAGGCAAAGTGGTATCTGAAGGCAATGGACATGTTGATAAGCTTTCACCGATATCCTC>1271
OsPCS1#3  1066>GAGGGGCTTAAAAATGTCATTACAGGCAAAGTGGTATCTGAAGGCAATGGACATGTTGATAAGCTTTCACCGATATCCTC>1145
OsPCS1#4  1116>GAGGGGCTTAAAAATGTCATTACAGGCAAAGTGGTATCTGAAGGCAATGGACATGTTGATAAGCTTTCACCGATATCCTC>1195
                        *         *         *         *         *         *         *         *

Genome    2561>GACTGAAACATGCTTCTGCAATTCAACTCTGAGCAATGAAA-CTGTC-AATTATCCATCAAACACAGAC-ATTCTAACTG>2637
CK083842   503>GACTGAAACATGCTTCTGCAATTCAACTCTGAGCAATGAAA-CTGTC-AATTATCCATCAAACACAGAC-ATTCTAACTG>579
CB646799   498<GACTGAAACATGCTTCTGCAATTCAACTCTGAGCAATGAAA-CTGTC-AATTATCCATCAAACACAGAC-ATTCTAACTG<422
C96924      32>-----AAACATGCTTCTGCAATNCACCTCTGAGCACTGAAACCTGTCAAATTATCCATCAAACACAGACCANTCTAACTG>106
OsPCS1#1  1442>GACTGAAACATGCTTCTGCAATTCAACTCTGAGCAATGAAA-CTGTC-AATTATCCATCAAACACAGAC-ATTCTAACTG>1518
OsPCS1#2  1272>GACTGAAACATGCTTCTGCAATTCAACTCTGAGCAATGAAA-CTGTC-AATTATCCATCAAACACAGAC-ATTCTAACTG>1348
OsPCS1#3  1146>GACTGAAACATGCTTCTGCAATTCAACTCTGAGCAATGAAA-CTGTC-AATTATCCATCAAACACAGAC-ATTCTAACTG>1222
OsPCS1#4  1196>GACTGAAACATGCTTCTGCAATTCAACTCTGAGCAATGAAA-CTGTC-AATTATCCATCAAACACAGAC-ATTCTAACTG>1272
                        *         *         *         *         *         *         *         *

Genome    2638>TTCTATTACTGTCGTTACATCCTAGCACATGGTTGTGCATTGAAGATGAGAAGTTGAAAGCTGAATTTCAGAGCCTTGTT>2717
CK083842   580>TTCTATTACTG~~~~~~~~~~~~~~~~~~~~~~~~~~~~~~~~~~~~~~~~~~~~~~~~~~~~~~~~~~~~~~~~~~~~~>590
CB646799   421<TTCTATTACTGTCGTTACATCCTAGCACATGGTTGTGCATTGAAGATGAGAAGTTGAAAGCTGAATTTCAGAGCCTTGTT<342
C96924     107>TTCTATTACTGTCGTTACATCCTAGCACATGGTTGTGCATTGAAGATGAGAAGTTGAAAGCTGAATTTCAGAGCCTTGTT>186
OsPCS1#1  1519>TTCTATTACTGTCGTTACATCCTAGCACATGGTTGTGCATTGAAGATGAGAAGTTGAAAGCTGAATTTCAGAGCCTTGTT>1598
OsPCS1#2  1349>TTCTATTACTGTCGTTACATCCTAGCACATGGTTGTGCATTGAAGATGAGAAGTTGAAAGCTGAATTTCAGAGCCTTGTT>1428
OsPCS1#3  1223>TTCTATTACTGTCGTTACATCCTAGCACATGGTTGTGCATTGAAGATGAGAAGTTGAAAGCTGAATTTCAGAGCCTTGTT>1302
OsPCS1#4  1273>TTCTATTACTGTCGTTACATCCTAGCACATGGTTGTGCATTGAAGATGAGAAGTTGAAAGCTGAATTTCAGAGCCTTGTT>1352
                        *         *         *         *         *         *         *         *

Genome    2718>TCTACGGACGATCTTCCTGATCCTCTTAAACTGGAGGTGTGCTGTCTCACTCCTAGAACCTTGCATTGATTGTTTACCTT>2797
CB646799   341<TCTACGGACGATCTTCCTGATCCTCTTAAACTGGAG--------------------------------------------<306
C96924     187>TCTACGGACGATCTTCCTGATCCTCTTAAACTGGAG-------------------------------------------->222
OsPCS1#1  1599>TCTACGGACGATCTTCCTGATCCTCTTAAACTGGAG-------------------------------------------->1634
OsPCS1#2  1429>TCTACGGACGATCTTCCTGATCCTCTTAAACTGGAGGTGTGCTGTCTCACTCCTAGAACCTTGCATTGA~~~~~~~~~~~>1497
OsPCS1#3  1303>TCTACGGACGATCTTCCTGATCCTCTTAAACTGGAGGTGTGCTGTCTCACTCCTAGAACCTTGCATTGA~~~~~~~~~~~>1371
OsPCS1#4  1353>TCTACGGACGATCTTCCTGATCCTCTTAAACTGGAGGTGTGCTGTCTCACTCCTAGAACCTTGCATTGA~~~~~~~~~~~>1421
                        *         *         *         *         *         *         *         *
                        *         *         *         *         *         *         *         *

Genome    3358>TCTATTCTAAGTAGAGCTACTATTTATATTGACAGATATTGCACCTAAGGAGGCAGCTCCGTTATCTGAAGGCTTGTAGA>3437
CB646799   305<-----------------------------------ATATTGCACCTAAGGAGGCAGCTCCGTTATCTGAAGGCTTGTAGA<261
C96924     223>-----------------------------------ATATTGCACCTAAGGAGGCAGCTCCGTTATCTGAAGGCTTGTAGA>267
OsPCS1#1  1635>-----------------------------------ATATTGCACCTAAGGAGGCAGCTCCGTTATCTGAAGGCTTGTAGA>1679
                        *         *         *         *         *         *         *         *

Genome    3438>GAAAAGGAGGCATATGAAGATACTTGGCCACAACCTTGGGAACAATGCTAACATGGCAACCCACTCACTTAATAATGAAT>3517
CB646799   260<GAAAAGGAGGCATATGAAGATACTTGGCCACAACCTTGGGAACAATGCTAACATGGCAACCCACTCACTTAATAATGAAT<181
C96924     268>GAAAAGGAGGCATATGAAGATACTTGGCCACAACCTTGGGAACAATGCTAACATGGCAACCCACTCACTTAATAATGAAT>347
OsPCS1#1  1680>GAAAAGGAGGCATATGAAGATACTTGGCCACAACCTTGGGAACAATGCTAACATGGCAACCCACTCACTTAATAATGAAT>1759
                        *         *         *         *         *         *         *         *
```

(B) *OsPCS2*

```
                        *         *         *         *         *         *         *         *
Genome      81>AGAGAAGCACGCATCCGATCGCCGGCGACGACCGAATCTGTGCCGGGCGGCAGGAAGGAGAGCGGCGATGGCAGCGATGG>160
OsPCS2#1    81>AGAGAAGCACGCATCCGATCGCCGGCGACGACCGAATCTGTGCCGGGCGGCAGGAAGGAGAGCGGCGATGGCAGCGATGG>160
OsPCS2#2    81>AGAGAAGCACGCATCCGATCGCCGGCGACGACCGAATCTGTGCCGGGCGGCAGGAAGGAGAGCGGCGATGGCAGCGATGG>160
AK071958     0>~~~~~~~~~~~~~~~~~~~~~~~~~~~~~~~~~~~~~~~~~~~~~~~~~~~~~~~~~~~~~~~~~~~~~~~~~~~~~~~~>0
OsPCS2#3     1>~~~~~~~~~~~~~~~~~~~~~~~~~~~~~~~~~~~~~~~~~~~~~~~~~~~~~~~~~~~~~~~~~~~ATGGCAGCGATGG>13
                        *         *         *         *         *         *         *         *
                        *         *         *         *         *         *         *         *
Genome     561>CTCTGCCTCCTCCCAGGACTGCCATCTCATCGCATCCTACCACAGGAAGCCTTTCAAACAGGTTGTTTTTGTTAATCCTT>640
OsPCS2#1   561>CTCTGCCTCCTCCCAGGACTGCCATCTCATCGCATCCTACCACAGGAAGCCTTTCAAACAG------------------->621
OsPCS2#2   561>CTCTGCCTCCTCCCAGGACTGCCATCTCATCGCATCCTACCACAGGAAGCCTTTCAAACAG------------------->621
AK071958     0>~~~~~~~~~~~~~~~~~~~~~~~~~~~~~~~~~~~~~~~~~~~~~~~~~~~~~~~~~~~~~~~~~~~~~~~~~~~~~~~~>0
OsPCS2#3   414>CTCTGCCTCCTCCCAGGACTGCCATCTCATCGCATCCTACCACAGGAAGCCTTTCAAACAG------------------->474
                        *         *         *         *         *         *         *         *
Genome     641>CTCTTCTTCATTTCAAGGTCTTAATAACCAACCAATTTATCATTGCATTGCATTTCAGACTGGAACCGGCCATTTCTCTC>720
OsPCS2#1   622>----------------------------------------------------------ACTGGAACCGGCCATTTCTCTC>643
OsPCS2#2   622>----------------------------------------------------------ACTGGAACCGGCCATTTCTCTC>643
AK071958     1>~~~~~~~~~ATTTCAAGGTCTTAATAACCAACCAATTTATCATTGCATTGCATTTCAGACTGGAACCGGCCATTTCTCTC>71
OsPCS2#3   475>----------------------------------------------------------ACTGGAACCGGCCATTTCTCTC>496
                        *         *         *         *         *         *         *         *
                        *         *         *         *         *         *         *         *
Genome     801>CCACTCCCACTGCTTTGGGAAGCCATGAATACAACTGATGACGCAACTGGTCTACTCAGGGGGTCTCTTCTCTACCCATC>880
OsPCS2#1   724>CCACTCCCACTGCTTTGGGAAGCCATGAATACAACTGATGACGCAACTGGTCTACTCAGGGG------------------>785
OsPCS2#2   724>CCACTCCCACTGCTTTGGGAAGCCATGAATACAACTGATGACGCAACTGGTCTACTCAGGGG------------------>785
AK071958   152>CCACTCCCACTGCTTTGGGAAGCCATGAATACAACTGATGACGCAACTGGTCTACTCAGGGG------------------>213
OsPCS2#3   577>CCACTCCCACTGCTTTGGGAAGCCATGAATACAACTGATGACGCAACTGGTCTACTCAGGGG------------------>638
                        *         *         *         *         *         *         *         *
                        *         *         *         *         *         *         *         *
Genome    4721>CCATCTTGATATTTGAAGGTTCATGCTTATCTCAAGGCACACTGCAGCTCCTTCATTGCTCTACACAGTGGTAAACATGC>4800
OsPCS2#1   786>------------------GTTCATGCTTATCTCAAGGCACACTGCAGCTCCTTCATTGCTCTACACAGTG---------->837
OsPCS2#2   786>------------------GTTCATGCTTATCTCAAGGCACACTGCAGCTCCTTCATTGCTCTACACAGTG---------->837
AK071958   214>------------------GTTCATGCTTATCTCAAGGCACACTGCAGCTCCTTCATTGCTCTACACAGTG---------->265
OsPCS2#3   639>------------------GTTCATGCTTATCTCAAGGCACACTGCAGCTCCTTCATTGCTCTACACA------------->687
                        *         *         *         *         *         *         *         *
Genome    4801>CAATGCTTGGTATATCGAATTCAAGTGGGTACATTGATATAATTTCCTTTATATCATTCCTTTAGCTTTTGTGCAGAGTT>4880
OsPCS2#1   838>----------------------------------------------------------------------------AGTT>841
OsPCS2#2   838>-----------------------------------------------------------------CTTTTGTGCAGAGTT>852
AK071958   266>----------------------------------------------------------------------------AGTT>269
OsPCS2#3   688>-----------------------------------------------------------------CTTTTGTGCAGAGTT>702
                        *         *         *         *         *         *         *         *
Genome    4881>GCAGAGATGAAAGCTGGAAAAGCATGGCGAAGTATTGCATGGAAGATGTACCCGATCTTCTTAAGGATGAGAGTGTAGAC>4960
OsPCS2#1   842>GCAGAGATGAAAGCTGGAAAAGCATGGCGAAGTATTGCATGGAAGATGTACCCGATCTTCTTAAGGATGAGAGTGTAGAC>921
OsPCS2#2   853>GCAGAGATGAAAGCTGGAAAAGCATGGCGAAGTATTGCATGGAAGATGTACCCGATCTTCTTAAGGATGAGAGTGTAGAC>932
AK071958   270>GCAGAGATGAAAGCTGGAAAAGCATGGCGAAGTATTGCATGGAAGATGTACCCGATCTTCTTAAGGATGAGAGTGTAGAC>349
OsPCS2#3   703>GCAGAGATGAAAGCTGGAAAAGCATGGCGAAGTATTGCATGGAAGATGTACCCGATCTTCTTAAGGATGAGAGTGTAGAC>782
                        *         *         *         *         *         *         *         *
                        *         *         *         *         *         *         *         *
Genome    5041>TAGGAGACAAGAGGAAGGAGGATCAGGATTAAGCAAAGAGGAGGAAGAAAGGCTTATTTTGAAGGTCATCTTCAACCTTT>5120
OsPCS2#1  1002>TAGGAGACAAGAGGAAGGAGGATCAGGATTAAGCAAAGAGGAGGAAGAAAGGCTTATTTTGAAG---------------->1065
OsPCS2#2  1013>TAGGAGACAAGAGGAAGGAGGATCAGGATTAAGCAAAGAGGAGGAAGAAAGGCTTATTTTGAAG---------------->1076
AK071958   430>TAGGAGACAAGAGGAAGGAGGATCAGGATTAAGCAAAGAGGAGGAAGAAAGGCTTATTTTGAAG---------------->493
OsPCS2#3   863>TAGGAGACAAGAGGAAGGAGGATCAGGATTAAGCAAAGAGGAGGAAGAAAGGCTTATTTTGAAG---------------->926
                        *         *         *         *         *         *         *         *
Genome    5121>TCTTTTCAATCTGGGATTCTGGATCAGAATTTTCTTTTTGTCTCTTTTAACACACCTTACACTGTGTGCACTTGCAGGAA>5200
OsPCS2#1  1066>-----------------------------------------------------------------------------GAA>1068
OsPCS2#2  1077>-----------------------------------------------------------------------------GAA>1079
AK071958   494>-----------------------------------------------------------------------------GAA>496
OsPCS2#3   927>-----------------------------------------------------------------------------GAA>929
                        *         *         *         *         *         *         *         *
                        *         *         *         *         *         *         *         *
Genome    5681>CTTCATGATGATCTTAAACGAGAGGTTTGTTGTATTAATCCAAACATCCCTTGCTCCCTACTCCCTAGCAAGATTTGTTT>5760
OsPCS2#1  1549>CTTCATGATGATCTTAAACGAGAG-------------------------------------------------------->1572
OsPCS2#2  1560>CTTCATGATGATCTTAAACGAGAG-------------------------------------------------------->1583
AK071958   977>CTTCATGATGATCTTAAACGAGAG-------------------------------------------------------->1000
OsPCS2#3  1410>CTTCATGATGATCTTAAACGAGAG-------------------------------------------------------->1433
                        *         *         *         *         *         *         *         *
                        *         *         *         *         *         *         *         *
Genome    6481>AAAAACATTTGTTGCACTAGTTTAGAATAACATTTTGTCTGTTGACACAGATATTGCATCTAAGACGGCAACTCCATTAT>6560
OsPCS2#1  1573>--------------------------------------------------ATATTGCATCTAAGACGGCAACTCCATTAT>1602
OsPCS2#2  1584>--------------------------------------------------ATATTGCATCTAAGACGGCAACTCCATTAT>1613
AK071958  1001>--------------------------------------------------ATATTGCATCTAAGACGGCAACTCCATTAT>1030
OsPCS2#3  1434>--------------------------------------------------ATATTGCATCTAAGACGGCAACTCCATTAT>1463
                        *         *         *         *         *         *         *         *
```

```
Genome     6561>GTGAGGTCCTGTAAAGAGGAGGAATATGGAGATCCTGTGCCACAATCCCATTAACAATGATGCAAATCGCGCAGTTGGTT>6640
OsPCS2#1   1603>GTGAGGTCCTGTAAAGAGGAGGAATATGGAGATCCTGTGCCACAATCCCATTAACAATGATGCAAATCGCGCAGTTGGTT>1682
OsPCS2#2   1614>GTGAGGTCCTGTAAAGAGGAGGAATATGGAGATCCTGTGCCACAATCCCATTAACAATGATGCAAATCGCGCAGTTGGTT>1693
AK071958   1031>GTGAGGTCCTGTAAAGAGGAGGAATATGGAGATCCTGTGCCACAATCCCATTAACAATGATGCAAATCGCGCAGTTGGTT>1110
OsPCS2#3   1464>GTGAGGTCCTGTAAAGAGGAGGAATATGGAGATCCTGTGCCACAATCCCATTAA~~~~~~~~~~~~~~~~~~~~~~~~~~>1517
                         *         *         *         *         *         *         *         *
```

Supplementary Fig. S1 Alignment of *OsPCS* sequences among genomic sequence, transcripts, and ESTs. (A) *OsPCS1* genomic sequence, ESTs (CB646798, CK083842, CB646799 and C96924) and transcript sequences (*OsPCS1*#1–4) were compared. *OsPCS1*#4 is the transcript sequence on the RAP-DB (AF439787). **(B)** *OsPCS2* genomic sequence and transcript sequences (*OsPCS2*#1–3) were compared. *OsPCS2*#2 (AK071754) and AK071958 are the transcript sequences on the RAP-DB. White boxes show the start codon and the stop codon. Gray boxes show the splice donor sites (GT) and the splice acceptor sites (AG). Black boxes show the site different from genomic sequence.

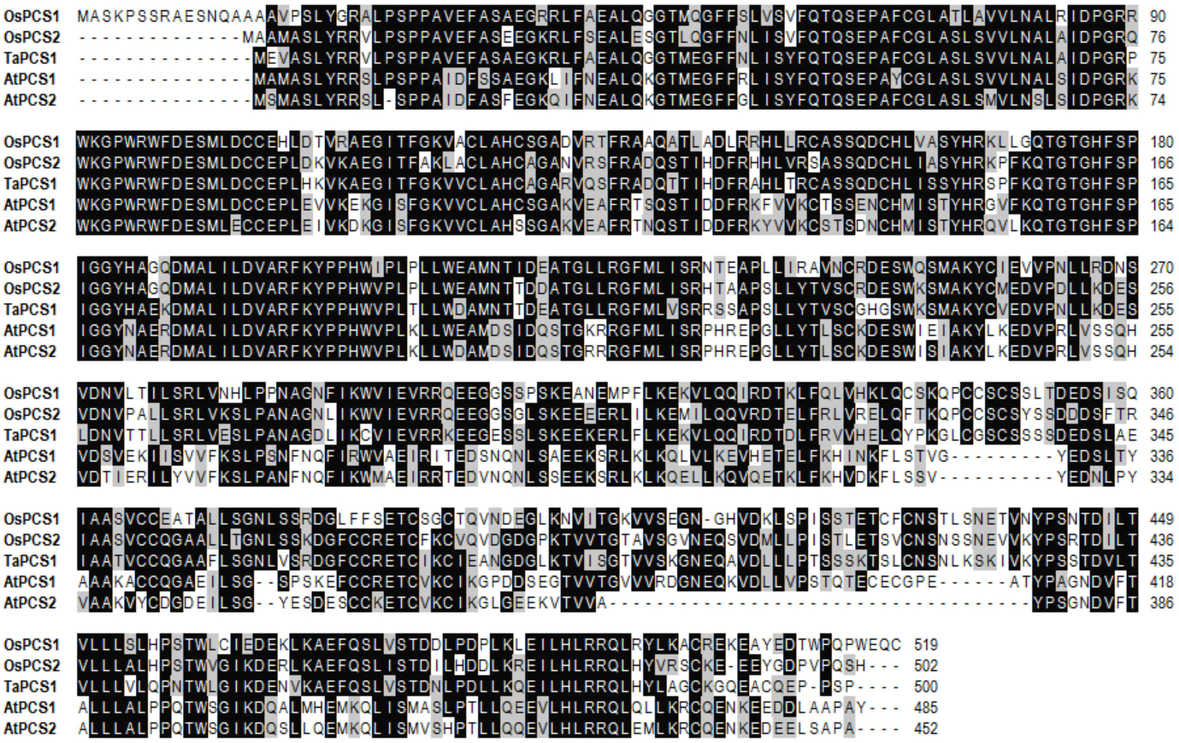

Supplementary Fig. S2 Alignment of predicted amino acid sequences of PC synthase from rice, *Arabidopsis*, and wheat. Black boxes show identical amino acid residues. Gray boxes show similar amino acid residues. Alignment was conducted by ClustalW in the DNA Data Bank of Japan (DDBJ). Accession number of the sequences is as following. AtPCS1: AAD50593, AtPCS2: AAK94671, TaPCS1: AAD50592.

```
OsPCS1          1 ------------------------------------------------AC      2
                                                                  |.
OsPCS2          1 GCATCTAAGACGGCAACTCCATTATGTGAGGTCCTGTAAAGAGGAGGAAT     50

OsPCS1          3 ATGG-------------CAA-CCCACTCACTTAATAATGAATTTCGCTGT     38
                  ||||             ||| ||||.|.||  |||.|||.|..||||   
OsPCS2         51 ATGGAGATCCTGTGCCACAATCCCATTAAC--AATGATGCAAATCGC---     95

OsPCS1         39 TCACTGGGCCTTTGGTTGCTGGCTTAACCTTACTGGAAATTGACGCGAAA     88
                         ||..||||||         |||   |||||    ||.||.|||
OsPCS2         96 -------GCAGTTGGTT---------ACC---CTGGA----GATGCAAAA    122

OsPCS1         89 AGAAAAGGTTAGGATGTTAGGAGAGGAGGTGAAAA------------TAT    126
                  |.||..||||           ||||||||    ||            |||
OsPCS2        123 AAAAGGGGTT-----------AGAGGAGG----AACTACATACTCCGTAT    157

OsPCS1        127 TACCTCCTTTTCAAATAAGGACTATATAAACAATTTTCTGATAATAATGA    176
                  |||||...||||.|.|.|||||             ||||.||        
OsPCS2        158 TACCTTTGTTTCGAGTGAGGAC-------------TTCTCAT--------    186

OsPCS1        177 TGTTTT--GGCAATTGGC--AAGTACTTTGCAACCGTGAAG-TATCTTTC    221
                    ||||  |.||..||.|  .|| ||   |.|.|||||.|| .||.||  
OsPCS2        187 --TTTTGAGACACCTGACCTGAG-AC---GGATCCGTGTAGACATGTT--    228

OsPCS1        222 CTTGCTTAATTTCTCTTTTGTT--TTTCCCTTGTACTTCTGATAGTTCCA    269
                  |.||.|.|   ||.|.|.||.|  ||||.|||||        ||||..||
OsPCS2        229 CATGTTCA---TCACCTGTGGTCGTTTCTCTTGT--------TAGTGACA    267

OsPCS1        270 --TGTACTTTATTCCCTCTAATTAATGGATAAGCACACTTAGCT------    311
                    || ||            ||.||..||..|.||||||   |||      
OsPCS2        268 ACTG-AC------------AACTAGCGGGGAGGCACAC---GCTAATTGT    301

OsPCS1        312 --------CGTCCT------------    317
                          .|||||
OsPCS2        302 GCGGCTGGTGTCCTTGCAAAAGTTTC    327
```

Supplementary Fig. S3 Alignment of RNAi trigger sequences of 3′-UTR in *OsPCS1* and *OsPCS2*.

A segment of 317 bp or 327 bp in the 3′ untranslated region specific to *OsPCS1* or *OsPCS2*, respectively, was used for the RNAi trigger. Global alignment of two nucleotide sequences was conducted by the EMBOSS Needle. Nucleotide sequence identity is 39.9%.

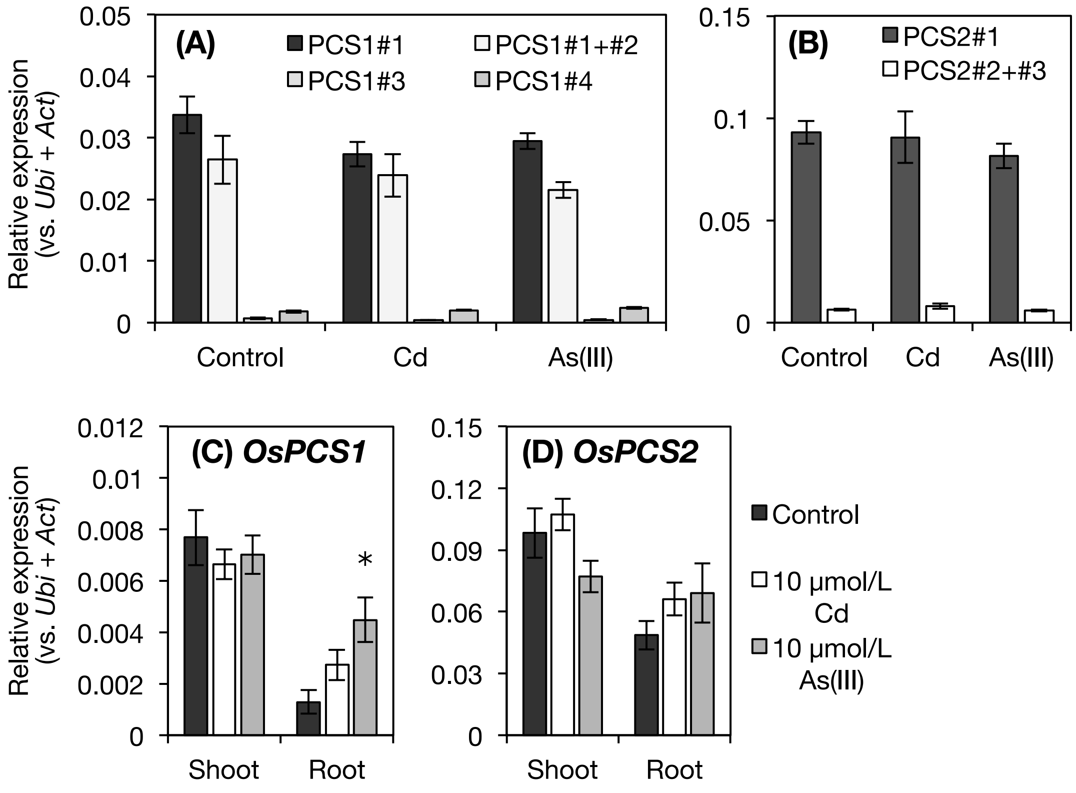

Supplementary Fig. S4 Expression levels of *OsPCS*. (A, B) Relative expression of *OsPCS1* and *OsPCS2* transcriptional variants in shoots under control conditions and heavy metal toxicity. Specific primers for each transcript were used. **(C, D)** Total expression levels of *OsPCS1* and *OsPCS2*. Common primers to all of the variants were used. The expression level was analyzed by quantitative RT-PCR in 10-day-old seedlings hydroponically grown under control, Cd stress (10 μmol L^{-1} $CdSO_4$), or As(III) stress (10 μmol L^{-1} $NaAsO_2$) condition. *Ubiquitin* and *Actin* were used as internal standards. Values are expressed as means ± SE (n = 4). An asterisk (*) shows a significant difference from the control ($p < 0.05$, Dunnett test).

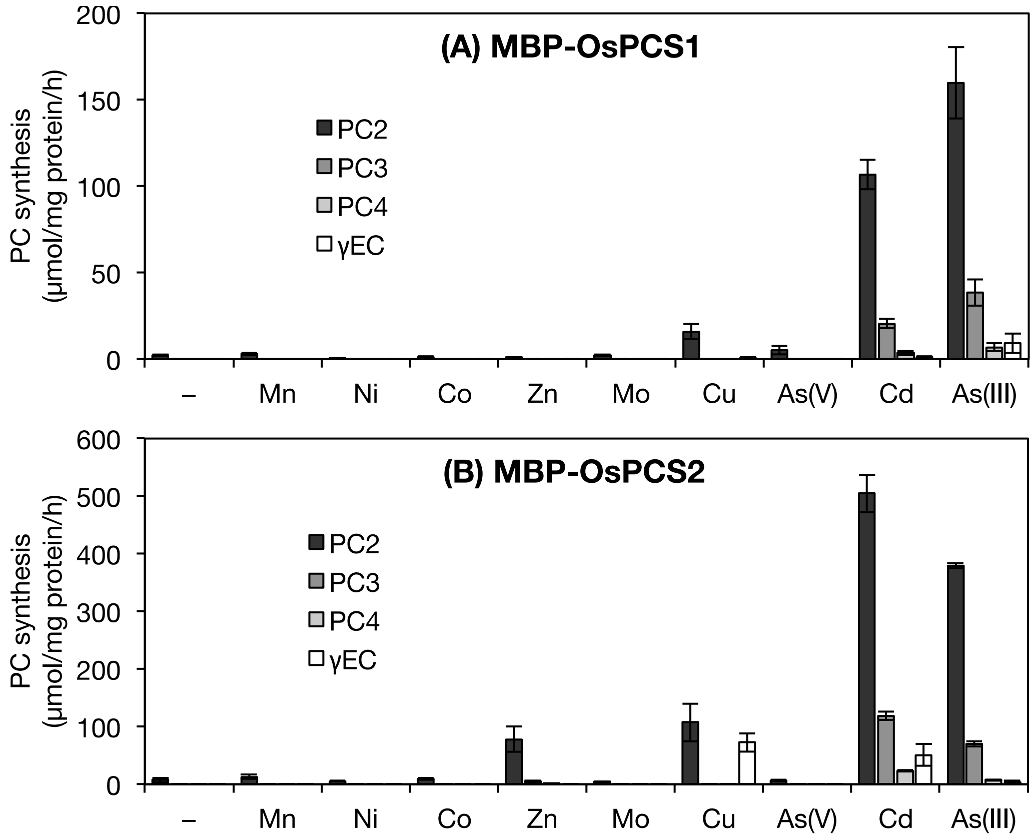

Supplementary Fig. S5 Activation of MBP-OsPCS by various heavy metals/metalloids. PC synthesis catalyzed by **(A)** OsPCS1 and **(B)** OsPCS2 in the presence of different heavy metals/metalloids. Purified MBP-OsPCS1 (15 µg mL^{-1}) or MBP-OsPCS2 (5 µg mL^{-1}) was incubated for 1 h in the reaction medium containing 10 mmol L^{-1} GSH, 200 mmol L^{-1} HEPES-KOH buffer (pH8.0), and 50 µmol L^{-1} of the metal ion indicated. The reaction was stopped by the addition of TFA at a final concentration of 1% (v/v). $PC_{2\text{–}4}$ and γEC were derivatized with monobromobimane and quantified by HPLC-fluorescent detection. Values are expressed as means ± SE from three independent experiments.

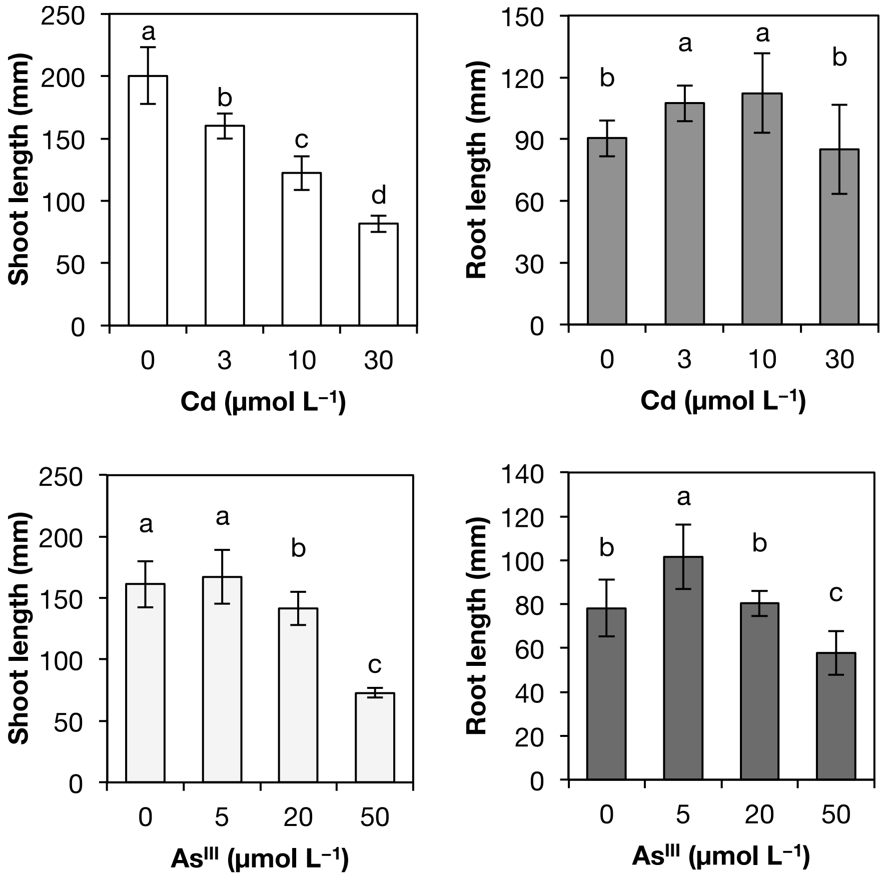

Supplementary Fig. S6 Growth of WT plants under Cd or As(III) stress.

Shoot and root lengths of WT plants under Cd or As(III) stress. WT plants (cv. Nipponbare) were hydroponically grown in the culture solution containing 3, 10, or 30 μmol L^{-1} Cd or 5, 20, or 50 μmol L^{-1} As(III) and the control culture solution for 10 days. Values are expressed as means ± SD (Cd: $n \geq 14$, As: $n \geq 10$). Different letters show a significant difference ($p < 0.05$, Tukey test).

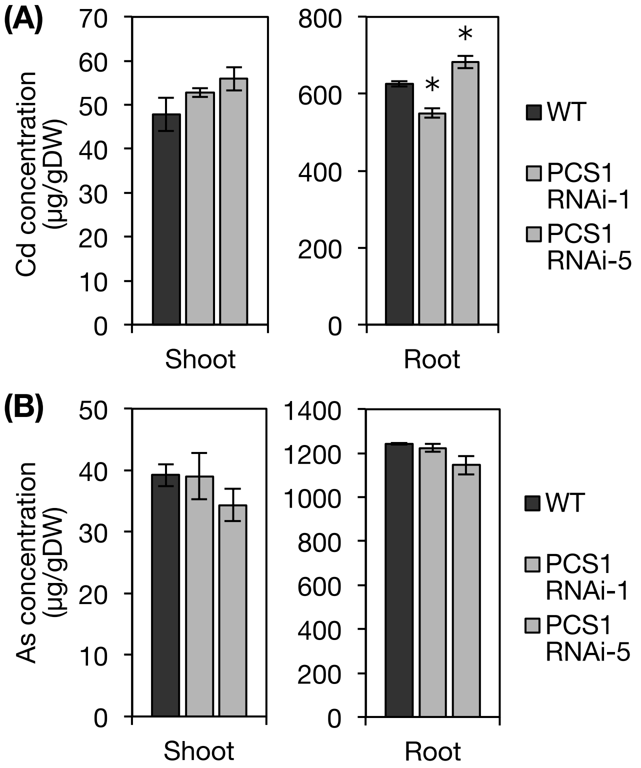

Supplementary Fig. S7 Cd and As concentrations in *OsPCS1* RNAi plants. (A) Cd concentration in shoots (left) and roots (right). **(B)** As concentration in shoots (left) and roots (right). WT and *OsPCS1* RNAi plants were hydroponically grown in a culture solution containing 10 µmol L^{-1} Cd or 20 µmol L^{-1} As(III) for 10 days. Each plant was separated into shoots and roots and oven-dried. After digestion with HNO_3 and $HClO_4$, Cd concentration was analyzed by atomic absorption spectroscopy and As concentration was analyzed by inductively coupled plasma atomic emission spectroscopy. Values are expressed as means ± SE (n = 4). An asterisk (*) shows a significant difference from WT plants ($p < 0.05$, Dunnett test).

Supplementary Table S1 Identity and similarity of predicted amino acid sequences of PC synthase from rice, *Arabidopsis*, and wheat.

Identity	OsPCS1	OsPCS2	AtPCS1	AtPCS2	TaPCS1
OsPCS1	-	69.6%	49.2%	46.9%	67.5%
OsPCS2	69.6%	-	56.5%	52.5%	76.3%
Similarity					
OsPCS1	-	80.4%	65.8%	62.5%	77.9%
OsPCS2	80.4%	-	71.2%	67.7%	86.7%

Global alignment of two protein sequences was conducted by EMBOSS Needle. Accession number of the sequences is as following. AtPCS1: AAD50593, AtPCS2: AAK94671, TaPCS1: AAD50592.

Supplementary Table S2 The concentration of thiol peptides in rice plants treated with BSO.

		Thiol peptides (nmol g^{-1}DW)						
−BSO		GSH	PC_2	PC_3	PC_4	hmGSH	γECE	γEC
Shoot	Control	48.0	-	-	-	25.3	-	-
	Cd	119	-	-	-	118	28.4	33.4
	As(III)	156	-	-	-	160	79.0	50.8
Root	Control	240	-	-	-	161	-	-
	Cd	188	50.4	53.8	5.80	790	221	208
	As(III)	444	532	203	11.0	1310	1260	1230
+BSO		GSH	PC_2	PC_3	PC_4	hmGSH	γECE	γEC
Shoot	Control	10.5	-	-	-	-	-	-
	Cd	8.84	-	-	-	-	-	-
	As(III)	21.1	-	-	-	6.24	4.96	12.2
Root	Control	7.04	-	-	-	-	-	-
	Cd	10.6	-	-	-	-	-	-
	As(III)	38.1	-	-	-	-	21.1	65.3

The 7-day-old seedlings of Nipponbare were transferred to a culture solution containing 10 μmol L^{-1} Cd or As(III) either alone or in combination with 0.5 mmol L^{-1} BSO. Controls without toxic metals were also tested. At 7 days after transfer, the concentration of thiol peptides in the shoots and roots was determined for a bulked sample of each treatment.

Supplementary Table S3 The sequences of primer used in this study.

For sequence analysis	
PCS1.seq1F	5′-CCAGACTGGAACAGGGCATT-3′
PCS1.seq2F	5′-GCCCTACCCTATCCAGCCTA-3′
PCS1.seqR	5′-AGGACGAGCTAAGTGTGCTT-3′
PCS1-2.3.R	5′-TCAATGCAAGGTTCTAGGAGTGAG-3′
PCS2.seqF	5′-AAGCAAGACGCGTCAAATCT-3′
PCS2.seq1R	5′-GAAACTTTTGCAAGGACACCA-3′
PCS2.seq2R	5′-TTTGACAACCTCGTTGCTTG-3′
For expression analysis	
PCS1-1.ex-F	5′-AACTGGAGATATTGCACCTAAGGA-3′
PCS1-1.ex-R	5′-GCCCAGTGAACAGCGAAA-3′
PCS1-1.2.ex-F	5′-GCATGGCAAAGTATTGCATAGAAG-3′
PCS1-1.2.ex-R	5′-TCCAGCATTGGGAGGAAGA-3′
PCS1-3.ex-F	5′-GGCTTCTCAGGGGTGGTAAA-3′
PCS1-4.ex-F	5′-CTTTATTGATCCGTGCAGTGGTA-3′
PCS1-3.4.ex-R	5′-ATCCTCCTTCCTCTTGTCTCCTAAC-3′
PCS2-1.ex-F	5′-CATTGCTCTACACAGTGAGTTGC-3′
PCS2-1.ex-R	5′-ATTTCACTAAGCGGGACAGAAG-3′
PCS2-2.ex-F	5′-TTTGGGAAGCCATGAATACAAC-3′
PCS2-2.ex-R	5′-TTTCATCTCTGCAACTCTGCAC-3′
Ubiquitin. F	5′-AGAAGGAGTCCACCCTCCACC-3′
Ubiquitin. R	5′-GCATCCAGCACAGTAAAACACG-3′
Actin1. F	5′-ATCCTTGTATGCTAGCGGTCGA-3′
Actin1. R	5′-ATCCAACCGGAGGATAGCATG-3′
PCS1.total.ex-F	5′-TCGCTTCAAATACCCTCCTCA-3′
PCS1.total.ex-R	5′-TCACTGCACGGATCAATAAAGG-3′
PCS2.total.ex-F	5′-CTTTGGGAAGCCATGAATACAAC-3′
PCS2.total.ex-R	5′-TTCCAGCTTTCATCTCTGCAAC-3′
For recombinant protein analysis	
PCS1.proF	5′-**ATG**GCGTCTAAACCAAGCAGC-3′
PCS1-1.proR	5′-ACTGCCTGCAGG**TTA**GCATTGTTCCCAAGGTTG-3′
PCS2.proF	5′-**ATG**GCAGCGATGGCATCCCT-3′
PCS2-1.proR	5′-GAGTCCTGCAGG**TTA**ATGGGATTGTGGCACAG-3′
For RNAi trigger	
PCS1.RNAi.F	5′-CACCACATGGCAACCCACTCACTT-3′
PCS2.RNAi.F	5′-CACCGCATCTAAGACGGCAACTCC-3′
PCS2.RNAi.R	5′-GAAACTTTTGCAAGGACACCA-3′
